# Harnessing Consumer Wearable Digital Biomarkers for Individualized Recognition of Postpartum Depression Using the All of Us Research Program Data Set: Cross-Sectional Study

**DOI:** 10.2196/54622

**Published:** 2024-05-02

**Authors:** Eric Hurwitz, Zachary Butzin-Dozier, Hiral Master, Shawn T O'Neil, Anita Walden, Michelle Holko, Rena C Patel, Melissa A Haendel

**Affiliations:** 1 Department of Genetics University of North Carolina at Chapel Hill Chapel Hill, NC United States; 2 School of Public Health University of California, Berkeley Berkeley, CA United States; 3 Vanderbilt Institute of Clinical and Translational Research Vanderbilt University Medical Center Nashville, TN United States; 4 International Computer Science Institute Berkeley, CA United States; 5 Department of Infectious Disease University of Alabama at Birmingham Birmingham, AL United States

**Keywords:** wearable device, All of Us, postpartum depression, machine learning, Fitbit, mobile phone

## Abstract

**Background:**

Postpartum depression (PPD) poses a significant maternal health challenge. The current approach to detecting PPD relies on in-person postpartum visits, which contributes to underdiagnosis. Furthermore, recognizing PPD symptoms can be challenging. Therefore, we explored the potential of using digital biomarkers from consumer wearables for PPD recognition.

**Objective:**

The main goal of this study was to showcase the viability of using machine learning (ML) and digital biomarkers related to heart rate, physical activity, and energy expenditure derived from consumer-grade wearables for the recognition of PPD.

**Methods:**

Using the *All of Us* Research Program Registered Tier v6 data set, we performed computational phenotyping of women with and without PPD following childbirth. Intraindividual ML models were developed using digital biomarkers from Fitbit to discern between prepregnancy, pregnancy, postpartum without depression, and postpartum with depression (ie, PPD diagnosis) periods. Models were built using generalized linear models, random forest, support vector machine, and k-nearest neighbor algorithms and evaluated using the κ statistic and multiclass area under the receiver operating characteristic curve (mAUC) to determine the algorithm with the best performance. The specificity of our individualized ML approach was confirmed in a cohort of women who gave birth and did not experience PPD. Moreover, we assessed the impact of a previous history of depression on model performance. We determined the variable importance for predicting the PPD period using Shapley additive explanations and confirmed the results using a permutation approach. Finally, we compared our individualized ML methodology against a traditional cohort-based ML model for PPD recognition and compared model performance using sensitivity, specificity, precision, recall, and *F*_1_-score.

**Results:**

Patient cohorts of women with valid Fitbit data who gave birth included <20 with PPD and 39 without PPD. Our results demonstrated that intraindividual models using digital biomarkers discerned among prepregnancy, pregnancy, postpartum without depression, and postpartum with depression (ie, PPD diagnosis) periods, with random forest (mAUC=0.85; κ=0.80) models outperforming generalized linear models (mAUC=0.82; κ=0.74), support vector machine (mAUC=0.75; κ=0.72), and k-nearest neighbor (mAUC=0.74; κ=0.62). Model performance decreased in women without PPD, illustrating the method’s specificity. Previous depression history did not impact the efficacy of the model for PPD recognition. Moreover, we found that the most predictive biomarker of PPD was calories burned during the basal metabolic rate. Finally, individualized models surpassed the performance of a conventional cohort-based model for PPD detection.

**Conclusions:**

This research establishes consumer wearables as a promising tool for PPD identification and highlights personalized ML approaches, which could transform early disease detection strategies.

## Introduction

### Background

Postpartum depression (PPD) is the most common complication of childbirth, occurring in approximately 1 in 7 women [1]. PPD can have several implications for women, manifesting in ways such as irritability, mood swings, fatigue, sleep and appetite disturbance, and thoughts of suicide [2]. Undetected PPD has also been shown to have financial implications for affected individuals as it can lead to challenges in maintaining employment or reduced work performance [3]. Furthermore, PPD has been linked to an elevated risk of mood disorders in the child as well as paternal depression [4,5].

Unfortunately, PPD remains significantly underdiagnosed and undertreated, as indicated by the strikingly low treatment rate of only 15% [6]. The current method of diagnosing PPD relies on screening instruments such as the Edinburgh Postnatal Depression Scale (EPDS), Center for Epidemiologic Studies Depression Scale, Patient Health Questionnaire, and Postpartum Depression Screening Scale, where the EPDS is the most commonly used instrument [7]. Often, women also need to undergo blood tests to assess thyroid function as the symptoms of PPD frequently overlap with hyperthyroidism [7]. Due to the challenges in diagnosing PPD, traditional approaches using these screening tools contribute to inadequate screening of women and subsequent underdiagnosis [8,9]. Therefore, the advent of new technologies is greatly needed to enable adequate and, hopefully, earlier detection of PPD.

Digital health tools have been gaining traction in recent years due to the near-ubiquitous ownership of smartphones [10]. Leveraging data passively collected by wearables (ie, digital biomarkers such as the average heart rate [HR], total steps, and calories burned per day) coupled with machine learning (ML) algorithms provides an opportunity to model the relationship between digital biomarkers and a particular disease for early recognition.

### Prior Work

Previous studies have demonstrated that ML algorithms using digital biomarkers from smartwatches can predict cardiovascular diseases, infection, diabetes, and mental health conditions [11-14]. For example, one study demonstrated that a wearable device could estimate the changes in the severity of patients with major depressive disorder, where their findings indicated that ML models exclusively using digital biomarkers from wearables achieved moderate performance with correlation coefficients of 0.56 (95% CI 0.39-0.73) and 0.54 (95% CI 0.49-0.59) in the time-split and user-split scenarios, respectively, between model predictions and actual Hamilton Depression Rating Scale scores [15]. Another study recruited individuals with moderate depression for 4 weeks to develop individualized ML models based on digital biomarkers to predict mood. Their findings displayed a correlation between digital biomarkers and depression, as evidenced by high-performing models with a mean absolute error of 0.77 (SD 0.27) points on the 7-point Likert scale, which corresponds to a mean absolute percent error of 27.9% (SD 10.3%) [16]. A study by Wang et al [17] found that students with higher depressive symptoms measured using the 8-item Patient Health Questionnaire were more likely to (1) use their phone at study locations (correlation coefficient [*r*]=0.39; *P*<.001) compared to all-day phone use (*r*=0.28; *P*=.01), (2) have irregular sleep time (*r*=0.30; *P*=.02) and wake time (*r*=0.27; *P*=.04) schedules, (3) be stationary for more time (*r*=0.37; *P*=.01), and (4) visit fewer places during the day (*r*=−0.27; *P*=.02). In addition, students with higher depressive symptoms measured using the 4-item Patient Health Questionnaire scores (1) were around a fewer number of conversations (*P*=.002), (2) slept for shorter durations (*P*=.02), (3) fell asleep later (*P*=.001), (4) woke up later (*P*=.03), and (5) visited fewer places (*P*=.003) over the previous 2-week period [17]. Other studies examining the association between digital biomarkers from wearables and depression include those by (1) Moshe et al [18], who demonstrated a negative association between the variability of locations visited and depressive symptoms (β=−.21; *P*=.04) and a positive association between total sleep time and time in bed and depressive symptoms (β=.24; *P*=.02); and (2) Rykov et al [19], who showed that a larger variation in nighttime HR between 2 AM and 4 AM (*r*=0.26; *P*=.001) and between 4 AM and 6 AM (*r*=0.18; *P*=.04) and lower regularity of weekday circadian activity based on steps (*r*=−0.17; *P*=.049) were associated with higher severity of depressive symptoms.

Additional research has been conducted related to understanding the relationship between wearable-derived digital biomarkers and PPD. For instance, one study showed that the features most predictive of maternal loneliness, which is commonly associated with PPD, were activity intensity, activity distribution during the day, resting HR, and HR variability [20]. It was also shown that women with milder depression symptoms typically had a larger daily radius of travel compared to those with more severe symptoms (2.7 vs 1.9 miles; *P*=.04) [21]. Finally, women with depression have been shown to have a lower HR variability (measured using the SD of 24-hour NN intervals, *F*=6.4; *P*=.01, and the SD of the averages of NN intervals in 5-minute segments, *F*=6.04; *P*=.02) and elevated HR while sleeping (*F*=5.05; *P*=.03) compared to women without depression [22].

While these studies highlight a relationship between digital biomarkers and depression or PPD, they suffer from the following limitations: (1) some studies use data in the model that need active patient engagement with partnered mobile apps, where user retention is known to decrease over time with health-related apps; (2) most studies do not use a predictive framework but rather examine the association between digital biomarkers and depressive symptoms; (3) only one study has developed individualized ML models; (4) most studies analyzing women with PPD have limited time frames and do not capture continuous longitudinal data across different phases of pregnancy; and (5) no studies have developed individualized ML models for women in the postpartum period combining data from wearables and the electronic health record (EHR) [23]. Therefore, a method that provides continuous and personalized monitoring without the need for clinical encounters to enable early detection of mental health disorders, including PPD, is needed.

### Goal of This Study

The *All of Us* Research Program (AoURP) is a comprehensive data set that collects several types of health-related data, including surveys, EHRs, physical measurements, and wearable data from Fitbit devices, with an emphasis on patient populations that have been previously underrepresented in biomedical research [24]. Currently, the longitudinal Fitbit data from >15,000 AoURP participants are made available to registered researchers on the *All of Us* Researcher Workbench, providing an opportunity to explore digital biomarkers in a diverse cohort of participants.

**Figure 1:**
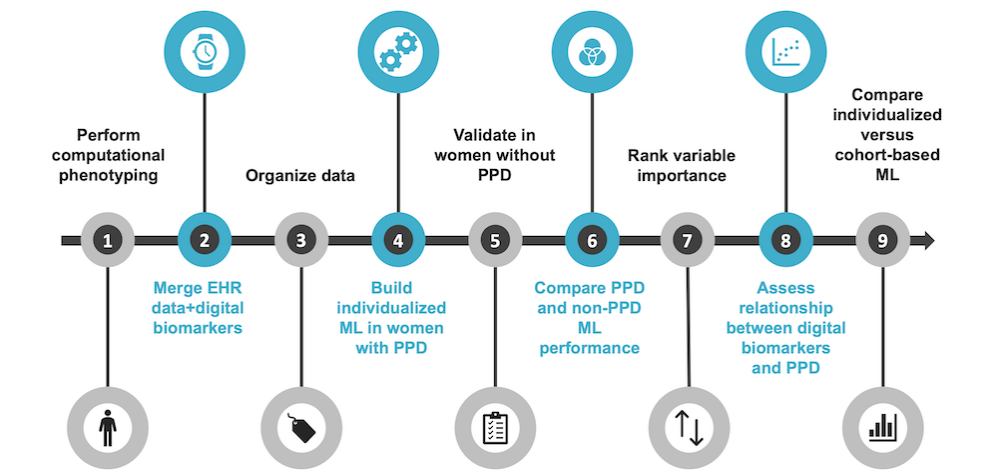
An overview of the analysis workflow to evaluate the potential for digital biomarkers in postpartum depression (PPD) recognition. (1) Develop and perform computational phenotyping of PPD and non-PPD cohorts; (2) merge with available digital biomarker data for each woman (heart rate, steps, physical activity, and calories burned); (3) classify each day as 1 of 4 periods (prepregnancy period, pregnancy, postpartum period without depression, or PPD); (4) build and assess individualized ML models testing random forest, generalized linear models, support vector machine, and k-nearest neighbor algorithms; (5) validate the machine learning (ML) approach in women without PPD; (6) compare individualized model performance in women with and without PPD; (7) determine variable importance for PPD recognition; (8) generate Shapley additive explanations dependence plots to assess the relationship between digital biomarkers and PPD; and (9) compare individualized ML models versus a cohort-based model for PPD detection. EHR: electronic health record.

It is unknown whether digital biomarkers from consumer wearables can be used to detect PPD. In this study, we combined several orthogonal approaches demonstrating that digital biomarkers can be used for individualized classification of PPD with data collected from Fitbit using the AoURP (Figure 1). This work demonstrated that (1) the integration of data sources, including EHR and wearable data, proves valuable for PPD recognition; (2) using longitudinal and continuous wearable data across various pregnancy phases supports ML model development; and (3) combining these integrated data sources facilitates the creation of individualized ML models, which may outperform cohort-based models. As such, our findings uncovered a novel method for recognizing PPD and serve as a framework that can be leveraged to facilitate early PPD detection. Moreover, the significance of this research underscores the promise of individualized ML models for detecting PPD, which can be applied to other mental health disorders.

## Methods

### Data Source and Platform

This study used the AoURP Registered Tier v6 data set. Study analysis was conducted using the AoURP Researcher Workbench cloud platform. All computational phenotyping, data processing, data analysis, and ML algorithms were conducted using R (R Foundation for Statistical Computing). Fitbit data collected in the AoURP adhere to a bring-your-own-device model, wherein participants who contribute their data are already in possession of a Fitbit device. The daily average HR, HR SD, minimum HR, quartile 1 HR, median HR, quartile 3 HR, and maximum HR were calculated using the Fitbit HR level table. The sum of steps was calculated using the Fitbit intraday steps table. Activity calories, calories burned during the basal metabolic rate (calories BMR), calories out, fairly active minutes, lightly active minutes, marginal calories, sedentary minutes, and very active minutes were taken from the Fitbit activity summary table. Day-level data were calculated for each of the 4 periods: prepregnancy period, pregnancy, postpartum period, and PPD (or PPD equivalent). All digital biomarkers included in this analysis are passively tracked by Fitbit; however, calories BMR is a calculated digital biomarker based on self-reported height, weight, age, and gender [25].

### Ethical Considerations

The protocol for the human participant research conducted was reviewed by the institutional review board of the AoURP (protocol 2021-02-TN-001). The institutional review board follows the regulations and guidance of the National Institutes of Health Office for Human Research Protections for all studies, ensuring that the rights and welfare of research participants are overseen and protected uniformly. Participants who contribute data to the AoURP have gone through an informed consent process with the option to withdraw at any time. Privacy is maintained by 1) storing data on protected computers, 2) researchers can’t see information to directly identify participants, such as name or social security number, 3) researchers sign a contract they won’t try to identify participants. Furthermore, the Researcher Workbench is only accessible to researchers through an institution with a signed Data Use Agreement and to researchers who complete the necessary training. If participants are asked (and decide) to go to an All of Us partner center for physical measurements to give blood, saliva, or urine samples, they are offered a one-time compensation of $25 in the form of cash, a gift card, or an electronic voucher.

In compliance with the Data and Statistics Dissemination Policy of the AoURP, counts of <20 cannot be presented to mitigate the risk of patient reidentification [26]. As the cohort of patients with PPD presented in this analysis comprised <20 patients, percentages were presented as percentage ranges (eg, instead of presenting the data as 53%, they were presented as 50%-55%). Publication of results in this manner has been approved by the AoURP Resource Access Board. Furthermore, race and ethnicity were not reported due to the limited sample size as requested by the AoURP Resource Access Board.

### Computational Phenotyping

#### Identifying Women With PPD

Women with PPD were identified using the following three-fold approach: (1) selecting women with a diagnosis of PPD using the condition data and identifying women with a record of (2) pregnancy or (3) delivery who had been diagnosed with depression or had antidepressant drug exposure during the postpartum period.

The first branch of the 3-fold approach to creating a cohort of women with PPD was conducted using Observational Medical Outcomes Partnership concept IDs in the condition table based on the Observational Health Data Sciences and Informatics initiative in Multimedia Appendix 1 [27,28]. For both the second and third branches of the method, we first identified women with a record of delivery (using condition data) or pregnancy (using the condition and survey tables) based on concept IDs from previously published work in Multimedia Appendix 1. Next, the data were filtered on the earliest record of delivery or pregnancy to capture and analyze digital biomarker data during the prepregnancy period. To estimate the date of pregnancy or delivery (depending on which was available for that individual), the date observed in the EHR from the AoURP was adjusted by adding or subtracting 9 months, which is a typical pregnancy duration [29]. Our next step was to estimate the window of the postpartum period, which was defined as starting from the date of delivery and spanning 24 months after that date, to monitor depressive symptoms [30,31]. Consistent with other EHR computational phenotyping studies of PPD, individuals were also classified as being PPD positive if they had a diagnosis of depression in the condition table or antidepressant drug exposure within the postpartum window [32] (Multimedia Appendix 1). Specific concepts containing the terms *episode*, *remission*, *reactive*, *atypical*, *premenstrual*, *schizoaffective*, and *seasonal* were excluded when identifying individuals with a depression diagnosis as they would not appropriately capture women with a persistent depression during the postpartum period. If a woman in the PPD cohort showed records of depression diagnosis and antidepressant drug exposure, we selected the earliest record to be considered the index date. For women with pregnancy and delivery data available, the index date and data used were based on the delivery record as this provided an elevated level of confidence in defining the postpartum period and, subsequently, whether the depression diagnosis or antidepressant drug exposure occurred during the postpartum period. Finally, the final PPD cohort was generated by selecting unique women from each of the 3 branches of our approach.

#### Identifying Women Without PPD

Women without PPD were selected as a control group to validate our approach because they experienced the same periods as women in the PPD cohort with the exception of having diagnosed or inferred PPD (see the previous section). Therefore, our modeling approach could be tested in an identical fashion (see more details about ML models in the section titled *Individualized ML Models for Women Without PPD*). To establish a cohort of women without PPD, we applied an identical rationale to that of the second and third branches of our PPD phenotyping, as described previously. Subsequently, women with records indicating PPD or depression diagnosis during the postpartum period from the condition table or any instances of antidepressant drug use from the drug exposure table were excluded.

### Data Preparation for Analysis and Individualized ML Models

To prepare the data for analysis and individualized ML models using wearable data, we first merged day-level data from Fitbit (HR, steps, physical activity, and calories burned; see Table S1 in Multimedia Appendix 2 [33-35] for more information on digital biomarkers) for each individual ranging from 2 years before to 30 days after the index date to capture their behavior before, during, and after pregnancy. Previous studies have demonstrated that HR, steps, and activity measurements from Fitbit are fairly accurate and can be used for research purposes [36,37]. The decision to choose measures related to HR instead of resting HR was based on the availability of data and the consideration of having enough measurements for each individual to train ML models. Digital biomarker data were filtered on days of *compliant* data, which were characterized by (1) at least 10 hours of Fitbit wear time within a day and (2) between 100 and 45,000 steps, as seen in previous studies [38]. Individuals from the PPD cohort were excluded from individualized ML models if they had <50 days of total data.

### Statistical Analysis

#### Assessing Variation in Digital Biomarkers Among Women

The *lme4* and *lmerTest* packages in R were used to construct hierarchical linear regression models aiming to assess the presence of noteworthy differences among women and examine the relationship between each period and digital biomarkers [39,40]. To assess whether there was a significant level of variation in digital biomarkers among individuals, we processed data to calculate the average value of each digital biomarker during each period (eg, average HR during the prepregnancy period, average HR during pregnancy, average HR during the postpartum period, and average HR during PPD) and conducted linear mixed-effects models with person ID as the random effect. One model was built for each digital biomarker, where the digital biomarker served as the outcome variable, the period was considered the independent variable, and person ID was incorporated as a random effect. The presence of significant variability among individuals was evaluated using the performance package at a significance level of .05 [41].

#### Interrupted Time-Series Analysis, Tukey Honest Significant Differences Test, and Digital Biomarker Directionality Assessment Between Periods

The interrupted time-series analysis (ITSA) was conducted using the *its.analysis* package in R with a significance level of .05 [42]. To compare whether there was a difference in digital biomarkers during different periods before, during, and after pregnancy, in addition to when patients experienced PPD, 4 periods were defined for each individual identified with PPD (prepregnancy period, pregnancy, postpartum period without depression [hereafter referred to as postpartum period], and postpartum period with depression [PPD]). The median duration of each period was 206 (IQR 154.50-313.50) days for the prepregnancy period, 258 (IQR 226-264) days for pregnancy, 42 (IQR 27.5-90) days for the postpartum period, and 42.5 (IQR 40.25-44.75) days for PPD. For each woman, a model was constructed for each digital biomarker, with 250 replications used for bootstrapping, which is a parameter of the *itsa.model()* function. Bootstrapping runs replications of the main model with randomly drawn samples and a trimmed median (10% removed); the *F* value is reported, and a bootstrapped *P* value is derived from it [42]. The dependent variable was the digital biomarker value, the *time* parameter was the date, and the interrupting variable was the period (prepregnancy period, pregnancy, postpartum period, and PPD). The mean and SD were calculated for each digital biomarker during each of the 4 periods for each woman. Furthermore, a Tukey honest significant difference (HSD) test was conducted to assess the statistical significance of the differences in each digital biomarker between each permutation of periods (PPD–prepregnancy period, PPD-pregnancy, PPD–postpartum period, postpartum period–prepregnancy period, postpartum period–pregnancy, and pregnancy–prepregnancy period) within each individual at a significance level of .05 [43]. Next, the percentage of women exhibiting a significant relationship was calculated for each digital biomarker in each group comparison (eg, PPD–prepregnancy period). To determine the overall trend in digital biomarker change between pairs of periods (eg, PPD and prepregnancy period, PPD and pregnancy, and PPD and postpartum period), the average difference across all individuals was computed for each digital biomarker. This average also included nonsignificant differences as they still contributed insights into the directionality of digital biomarkers during those periods even if the differences were not statistically significant. Finally, a 2-sided unpaired *t* test (2-tailed) at a significance level of .05 was conducted to assess the statistical significance of the net difference compared to 0, with positive change defined as an average value of >0 and negative change defined as an average value of <0. The outcomes were visualized in a heat map using the *ggplot2* package in R. Percentages were represented as percentage ranges to preserve patient confidentiality, with the upper value of each range depicted in the heat maps (eg, 62% would fall within the 60%-65% range, and 65% would be displayed in the heat map).

#### Evaluating Health Care–Seeking Behavior

Health care–seeking behavior was assessed by looking at the number of visits recorded for each woman during the postpartum period (ie, ranging from the date of delivery to 30 days after the index date for each woman). The number of visits was determined by counting the number of rows in the visit occurrence table in the AoURP. We subsequently conducted an unpaired 2-sided Wilcoxon test with a significance level of .05 to determine whether the medians exhibited a significant difference between the PPD and non-PPD cohorts.

We also examined the proportion of women who adhered to the recommendation set by the American College of Obstetricians and Gynecologists, which advised women to attend at least one visit within the initial 6 weeks of the postpartum period. Of note, this guideline was updated in 2018 and now recommends a postpartum visit within the first 3 weeks following delivery [33]. However, we used the pre-2018 guideline in our analysis because the AoURP cohort includes individuals enrolled before 2018. The percentages of women who attended postpartum visits within the first 6 weeks in the PPD and non-PPD cohorts were compared using a 2-proportion *z* test at a significance level of .05. The exact percentage of women in the PPD cohort, in addition to the exact counts used to calculate the percentages, was obfuscated to maintain patient privacy.

#### Comparing Self-Reported and Gold-Standard Weight Measurements

Weight measurements were queried in AoURP using the measurements table (Observational Medical Outcomes Partnership concept ID 3025315). Self-reported and gold-standard weight measurements were distinguished by referencing the *src_id* column, indicating a physical measurement (self-reported) as opposed to measurements obtained from an EHR site (gold standard). Subsequently, we identified the self-reported and gold-standard weight measurements with the shortest time interval for each woman. Only measurements taken within a period of <30 days were considered to ensure that the measurements were closely aligned and not too distant. The median and IQR of self-reported and gold-standard measurements were calculated and compared using a paired 2-sided Wilcoxon test at a significance level of .05. This process was repeated in the PPD and non-PPD cohorts.

#### Comparing Weight Across Periods of Pregnancy

Weights across different periods of pregnancy (prepregnancy period, pregnancy, postpartum period, and PPD [or PPD equivalent for those without PPD]) were computed in the PPD and non-PPD cohorts using linear mixed-effects models in the *lme4* package in R, with weight serving as the outcome variable, period as the independent variable, and person ID as the random effect. The results were evaluated at a significance level of .05. For women in the PPD cohort, the PPD period was used as the reference as it was the period of interest for understanding weight change. Similarly, the PPD-equivalent period was used as the reference for women in the non-PPD cohort. We further calculated the estimated means of weight across periods using the *emmeans* package in R for both the PPD and non-PPD cohorts.

#### Comparing Weight Retention in the PPD and Non-PPD Cohorts

To assess weight retention among women who experienced PPD compared to those without PPD, we first calculated the median weight of each woman during the prepregnancy period. Second, we identified the weight measurement during the postpartum period that was closest in value to the median prepregnancy weight on an individual basis. Third, the time difference in days was computed between the date of the weight measurement and the onset of pregnancy for each individual. Finally, we determined the median and IQR for the time difference in days mentioned in step 3 (ie, difference in days between the date of the weight measurement during the postpartum time period that was closest in value to the median prepregnancy weight for each individual) and subsequently conducted an unpaired 2-sided Wilcoxon test to assess the difference in medians at a significance level of .05 between women in the PPD and non-PPD cohorts.

### Building ML Models

#### Individualized ML Models for Women in the PPD Cohort

Individualized ML models were developed with the objective of determining the potential of digital biomarkers to differentiate among 4 distinct pregnancy phases: prepregnancy period, pregnancy, postpartum period without depression (ie, postpartum period), and postpartum period with depression (ie, PPD). Specifically, we sought to assess whether we could develop ML models for each woman to make a prediction to classify a day of Fitbit data as falling during the prepregnancy, pregnancy, postpartum, or PPD period based on behavioral and biometric data captured by digital biomarkers on Fitbit. In other words, the models tested whether there was a unique digital signature associated with each period of pregnancy in an individualized manner. Therefore, multinomial models were developed with period as the outcome with all 16 digital biomarkers as the features in the model (see Table S1 in Multimedia Appendix 2 for a list of the digital biomarkers included). Initially, our intention was to examine the model’s capacity to discriminate between periods with and without PPD, thereby constructing binomial classification models. However, we recognized the hierarchical nature of the data with repeated measurements (multiple days of data) during the prepregnancy, pregnancy, and postpartum time frames. Consequently, due to the repetitive nature of our data, we opted for constructing multinomial ML models to effectively discern among the 4 identified periods, where the PPD period was treated as both a period and a diagnosis. We were then able to focus on the PPD period by (1) constructing a confusion matrix to assess model performance for the PPD period at an individual level and (2) performing variable importance (see the following *Variable Importance* sections) for the PPD period.

To build intraindividual models, the data were filtered on each woman, where they were considered PPD negative ranging from 2 years before to 15 days before the index date and PPD positive from 14 days before to 30 days after the index date. We selected 14 days preceding the index date as the first day of being positive for PPD because the criteria for diagnosis state that patients must display 5 depressive symptoms lasting 2 weeks [44]. The time frame of 30 days following the index date was chosen because some individuals in the PPD cohort received antidepressant medication on the day of their diagnosis, which can begin to take effect after approximately 4 weeks of use [45]. For each individual, the data were centered and scaled before building models using 3 repeats of 10-fold cross-validation and a tune length of 5 with random forest (RF), generalized linear models (GLMs), support vector machine (SVM), and k-nearest neighbor (KNN) as these algorithms have been used in previous studies assessing depression using wearables [15,46]. Of note, no bootstrapping was performed as part of the individualized ML workflow. Models were built using the *Caret* package in R and evaluated using a combination of the κ statistic and multiclass area under the receiver operating characteristic curve (mAUC), which are standard metrics for classification ML models [47-50]. Model performance for each period was further assessed using a confusion matrix, which calculated sensitivity, specificity, precision, recall, and *F*_1_-score [50].

#### Comparing Individualized ML Model Performance Between Women With a History of Depression Before or During Pregnancy

To initially ascertain the presence of depression history before or during pregnancy within the PPD cohort, we determined the date of delivery (using condition data) or the date of pregnancy (using condition and survey data) based on the concept IDs detailed in Multimedia Appendix 1. Depending on the available data for each woman, the date of pregnancy was calculated by subtracting 9 months from the date of delivery, whereas the date of delivery was calculated by adding 9 months to the date of pregnancy, representing a standard pregnancy duration [29]. In cases in which both delivery and pregnancy records existed, priority was given to the date of delivery due to its heightened reliability.

For the evaluation of individualized ML model performance within the PPD cohort concerning women with a history of depression, the cohort was categorized into four subgroups encompassing (1) no previous depression history, (2) depression before pregnancy, (3) depression during pregnancy, and (4) depression both before and during pregnancy. To examine potential disparities in individualized ML model performance, a 2-sided unpaired *t* test was conducted with a significance threshold of .05. This analysis was executed to compare the no-depression-history group with the groups of women exhibiting depression before, during, or both before and during pregnancy. Sensitivity, specificity, precision, recall, and *F*_1_-score metrics were subjected to this statistical comparison process.

#### Individualized ML Models for Women Without PPD

To construct individualized ML models for women in the non-PPD cohort, we implemented an analogous approach to the one used for women in the PPD cohort, where an ML model was built for each woman with *period* as the multinomial outcome. It is worth noting that women without PPD would not have a fourth period (ie, postpartum period with depression in women with PPD) as they did not experience PPD. To ensure comparability and effectively gauge model performance between women with and without PPD, we created a PPD-equivalent period for the non-PPD cohort mirroring the PPD period. Considering that the median time to diagnose PPD was found to be 83 days following delivery, we ensured uniformity by setting the index date of the PPD-equivalent period at 83 days after delivery. As we established an index date aligned with that of the PPD cohort, the interval of 14 days before the index date was not considered as the PPD-equivalent period for these women because they did not actually experience PPD. The goal was to validate any observed alterations in the PPD cohort by investigating whether there were any changes in the digital signature between the postpartum and PPD-equivalent periods, which should not exist given that these women did not experience PPD. Subsequently, individualized ML models were constructed in a manner akin to those in the PPD cohort using the RF algorithm (as this algorithm yielded optimal results in the PPD cohort) using 3 repetitions of 10-fold cross-validation and a tuning length of 5. Similar to the approach developed for women in the PPD cohort, model performance was evaluated using sensitivity, specificity, precision, recall, and *F*_1_-score [49,50]. Models were not assessed using mAUC or κ as model performance only decreased in the PPD-equivalent period and not in the prepregnancy, pregnancy, or postpartum periods compared to those in the PPD cohort.

#### Comparing Individualized ML Model Performance for Women in the PPD and Non-PPD Cohorts

For comparing the performance of individualized ML models in the PPD cohort to those in the non-PPD cohort, we performed a 2-sided unpaired *t* test with a significance level of .05.

### Variable Importance

#### Shapley Additive Explanations Approach

We used the RF ML models to generate a ranking of digital biomarkers for each individual as these models had the best performance. Following that, Shapley values were computed for each measurement within each individualized model for the PPD class using the *iml* package in R [51]. To determine the feature ranking within individual models, we computed the average absolute Shapley values across all measurements for each digital biomarker and sorted the rankings from largest to smallest. We then tallied the number of models in which each biomarker ranked among the top 5 most predictive for the PPD class to produce an overall ranking of digital biomarkers. Furthermore, we determined the most predictive feature of PPD by totaling the number of models in which each digital biomarker ranked as the top predictor for the PPD class.

#### Permutation Approach

To enhance the robustness of our approach, variable importance was also computed using a permutation-based method in the *Caret* package in R [50]. Subsequently, the features were sorted based on the magnitude of values assigned for the variable importance regarding the PPD class. Using a similar methodology as with Shapley additive explanations (SHAP), we tabulated the number of models in which each digital biomarker ranked among the top 5 most predictive for the PPD class, yielding a comprehensive ranking of digital biomarkers. The frequency with which each feature ranked as the foremost predictive digital biomarker was also recorded for the PPD class.

### SHAP Dependence Plots

SHAP dependence plots were generated using the *gpplot2* package in R [52]. For each individual, plots were generated by graphing the Shapley value against the corresponding actual value for the digital biomarker. Given that the outcome of the models was multinomial (prepregnancy period, pregnancy, postpartum period, or PPD), 3 separate SHAP dependence plots were generated for each individual using calories BMR data during PPD with one other period (ie, one plot for the prepregnancy and PPD periods [referred to as prepregnancy vs PPD], one plot for pregnancy and PPD [referred to as pregnancy vs PPD], and one plot for the postpartum and PPD periods [referred to as postpartum vs PPD]) to more easily analyze the relationship between calories BMR in a binomial context between PPD and one other period. This process was repeated for women in the non-PPD cohort in a similar fashion to those in the PPD cohort, specifically, PPD-equivalent versus prepregnancy period (prepregnancy vs PPD-equivalent), PPD-equivalent versus pregnancy (pregnancy vs PPD-equivalent), and PPD-equivalent versus postpartum (postpartum vs PPD-equivalent). The Pearson correlation coefficient and its corresponding *P* value were computed at a significance level of .05, followed by calculating the percentages of women with and without a significant correlation. If a significant correlation was observed, we further determined its direction (positive or negative) and calculated the percentages of women with a positive or negative correlation. The overall consensus regarding the relationship was determined by comparing the percentage of positive and negative correlations for each digital biomarker across all individuals, thereby identifying which direction had a greater rate. In cases in which the proportion of women with a significant correlation was <40%, the direction was not assessed due to the small sample size, which may not be representative of the population.

### Building an ML Model for PPD Using a Cohort-Based Approach

For the construction of an ML model that assessed whether a woman had PPD, our focus was on using the PPD and PPD-equivalent periods sourced from both the PPD and non-PPD cohorts. We proceeded to develop a binomial RF classification model in which 75% of individuals from each cohort were designated for the training set and the remaining 25% were assigned to the test set using the *Caret* package in R [50]. To ensure the reliability of model performance assessment, we diligently executed train and test set divisions based on individual person IDs, thereby preventing any overlap of women between the 2 sets that could potentially distort the results [53]. The model’s target outcome pertained to a binary classification of whether an individual exhibited PPD relying on all 16 digital biomarkers as input (refer to Table S1 in Multimedia Appendix 2 for a comprehensive description of the digital biomarkers used). The data were normalized through centering and scaling procedures. Notably, repeated cross-validation was omitted due to the presence of repeated measurements stemming from various person IDs. The model’s construction integrated a tune length of 5. The models were evaluated using the same κ and area under the receiver operating characteristic curve metrics (not multiclass in this instance as the outcome was binary). Subsequently, a confusion matrix was generated to calculate sensitivity, specificity, precision, recall, and *F*_1_-score [47-50].

## Results

### Descriptive Statistics

**Figure 2:**
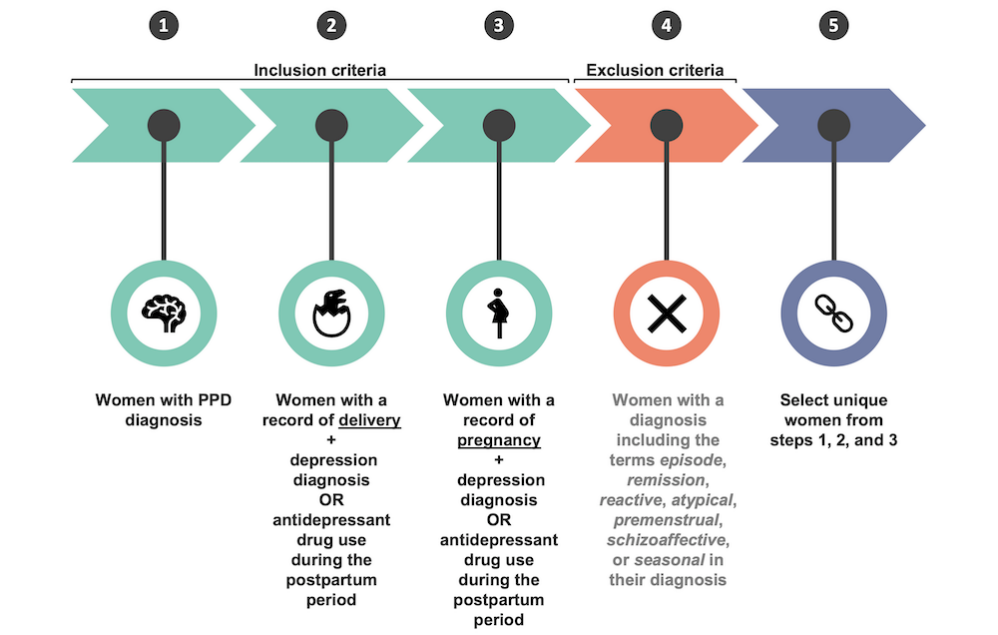
A schematic of postpartum depression (PPD) computational phenotyping.

Through computational phenotyping in the AoURP, a patient cohort of women who gave birth with PPD (n<20) and without PPD (n=39) provided valid Fitbit data (Figure 2). The median age in the PPD cohort was 35.60 (IQR 32.83-37.36) years compared to that in the non-PPD cohort, which was 33.60 (IQR 30.72-35.56) years. The median and IQR were calculated for each digital biomarker across all women in the PPD and non-PPD cohorts (Table 1). In both the PPD and non-PPD cohorts, we computed the median number of days with digital biomarker data during the prepregnancy, pregnancy, postpartum, and PPD (or PPD-equivalent) periods and the corresponding IQRs (additional details about the PPD-equivalent period, a similar fourth period for those without PPD, can be found in the *Methods* section; Table 1). Briefly, the digital biomarkers included in this analysis were daily average HR, HR SD, minimum HR, quartile 1 HR, median HR, quartile 3 HR, maximum HR, sum of steps, activity calories, calories BMR, calories out, fairly active minutes, lightly active minutes, marginal calories, sedentary minutes, and very active minutes (see the descriptions in Table S1 in Multimedia Appendix 2).

**Table 1:** Descriptive statistics of the postpartum depression (PPD) and non-PPD patient cohorts in the All of Us Research Program.

Descriptive statistics		PPD (n<20), median (IQR)	Non-PPD (n=39), median (IQR)
Age (y)		35.60 (32.83-37.36)	33.60 (30.72-35.56)
**Digital biomarker**			
	Average HR^a^ (bpm)	74.23 (68.36-80.66)	78.31 (72.32-83.97)
	HR SD (bpm)	12.18 (10.58-14.05)	12.70 (10.72-15.12)
	Minimum HR (bpm)	54.00 (49.00-60.00)	57.00 (52.00-61.00)
	Quartile 1 HR (bpm)	64.00 (59.00-71.00)	68.00 (62.00-74.00)
	Median HR (bpm)	72.00 (66.00-78.00)	76.00 (70.00-82.00)
	Quartile 3 HR (bpm)	81.00 (74.00-88.00)	85.00 (78.00-92.00)
	Maximum HR (bpm)	124.00 (117.00-135.00)	127.00 (119.00-141.00)
	Sum steps	7567.50 (4884.00-10536.25)	7352.00 (4838.00-10834.00)
	Activity calories	989.00 (742.75-1263.00)	964.00 (684.00-1275.00)
	Calories burned during BMR^b^	1466.00 (1379.00-1539.00)	1390.00 (1340.00-1496.00)
	Calories out	2236.00 (2012.00-2483.25)	2180.00 (1925.00-2465.50)
	Fairly active minutes	9.00 (0.00-24.00)	8.00 (0.00-23.00)
	Lightly active minutes	245.00 (189.00-315.00)	245.00 (187.50-310.00)
	Marginal calories	501.00 (349.00-665.00)	489.00 (322.00-680.00)
	Sedentary minutes	646.00 (563.00-741.00)	710.00 (607.00-880.50)
	Very active minutes	2.00 (0.00-18.00­)	4.00 (0.00-21.00)
**Number of days in each period**			
	Prepregnancy period	206.00 (154.50-313.50)	227.00 (109.50-340.75)
	Pregnancy	258.00 (226.00-264.00)	221.00 (129.00-269.50)
	Postpartum period	42.00 (27.50-90.00)	72.00 (46.00-82.00)
	PPD	42.50 (40.25-44.75)	29.00 (14.50-31.00)

^a^HR: heart rate.

^b^BMR: basal metabolic rate.

### Digital Biomarker Comparison Across Periods of Pregnancy Revealed Altered Profiles and Heterogeneity Among Women

Because of the known heterogeneity in depressive symptoms, we hypothesized that variability in digital biomarkers may exist across individuals in the PPD cohort [54]. To test this hypothesis, we conducted linear mixed-effects models for each digital biomarker in women with PPD, where we found that the random effect of person ID was significant (*P*<.001) for all digital biomarkers, suggesting meaningful variability across individuals (Table S2 in Multimedia Appendix 2). These results, coupled with a smaller cohort sample size, prompted us to perform subsequent analyses using an intraindividual approach.

**Figure 3:**
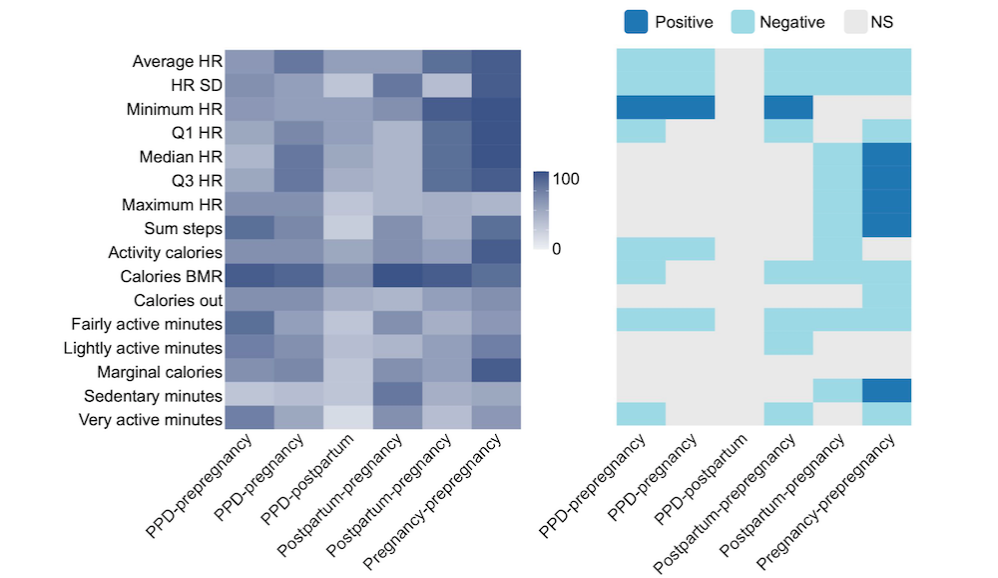
Digital biomarkers vary across different periods of pregnancy among women with postpartum depression (PPD). The percentage of women in the PPD cohort exhibiting a significant difference in digital biomarker values between each pair of periods (left [represented by 0-100]) and the direction of their relationship (right). The x-axis illustrates a comparison of Tukey honest significant differences (HSD) between 2 periods of interest, representing the subtraction of digital biomarker values between the first and second periods. Tukey HSD tests were individually conducted for each woman’s data, and the percentage showing a significant relationship was calculated and presented on the heat map. The heat map on the right illustrates the overall relationship between the digital biomarker during the 2 periods of interest among the women who exhibited a significant relationship (as indicated by the percentage shown on the left heat map), with the period listed second serving as the reference. In summary, the findings indicated that digital biomarkers undergo significant alterations across different periods of pregnancy on an individual basis. Calories BMR: calories burned during the basal metabolic rate; HR: heart rate; NS: not significant; Q1: quartile 1; Q3: quartile 3.

In women with PPD, we next sought to compare whether there was a difference in digital biomarkers across different periods of pregnancy: prepregnancy period, pregnancy, postpartum period, and PPD (where PPD represents both a period and a diagnosis). Therefore, an intraindividual ITSA and Tukey HSD test were conducted for each digital biomarker. Because of the physiological changes associated with pregnancy, such as increases in blood and stroke volume, in addition to the behavioral fluctuations that occur during PPD, such as a loss of energy and psychomotor retardation, we hypothesized that all digital biomarkers (those related to HR, steps, physical activity, and calories burned) would be altered across the prepregnancy, pregnancy, postpartum, and PPD periods [44,55-57]. ITSA results supported our hypothesis and demonstrated a significant difference in all digital biomarkers across periods in most women with PPD (Table S3 in Multimedia Appendix 2). Consistent with ITSA findings, Tukey HSD results showed that several digital biomarkers were significantly altered between PPD and other periods (prepregnancy, pregnancy, and postpartum periods; Figure 3). We further observed various trends in digital biomarkers between pairs of periods (ie, PPD and prepregnancy period, PPD and pregnancy, and PPD and postpartum period; Figure 3 and Table S4 in Multimedia Appendix 2).

### Individualized ML Models Effectively Differentiated PPD From Alternative Periods of Pregnancy

Having seen that digital biomarkers were significantly altered across multiple periods of pregnancy in women with PPD, we surmised that individualized multinomial ML models could accurately distinguish between our 4 periods of pregnancy (prepregnancy period, pregnancy, postpartum period, or PPD; Figure 3 and Tables S3 and S4 in Multimedia Appendix 2). Therefore, we sought to assess whether ML models for each woman could accurately classify an unknown day of Fitbit data as falling during the prepregnancy, pregnancy, postpartum, or PPD period based on behavioral and biometric data captured by digital biomarkers on Fitbit. In essence, the models examined whether there existed a distinct digital signature linked to each pregnancy period in an individualized fashion. To probe this hypothesis, intraindividual ML models were generated using RF, GLM, SVM, and KNN to conclude which algorithm would yield the best-performing results. Models were assessed using a combination of the mAUC and κ, which are 2 frequently used metrics [48,58]. After averaging the mAUC for individual models within each algorithm, the results revealed that RF models performed the best, followed by GLM, SVM, and then KNN, with an average mAUC of 0.85, 0.82, 0.75, and 0.74, respectively (Table 2). Assessing models in a similar fashion using another metric, κ, yielded concordant results for RF (0.80), GLM (0.74), SVM (0.72), and KNN (0.62) model performance, suggesting that the RF algorithm had the best performance and should be used going forward (Table 2).

**Table 2:** Individualized random forest (RF) models exhibited the best performance for multinomial period classification.

Algorithm	mAUC^a^, mean (SD)	κ, mean (SD)
Random forest	0.85 (0.09)	0.80 (0.15)
Generalized linear model	0.82 (0.09)	0.74 (0.16)
Support vector machine	0.75 (0.10)	0.72 (0.16)
k-nearest neighbor	0.74 (0.10)	0.62 (0.19)

^a^mAUC: multiclass area under the receiver operating characteristic curve.

As our analysis aimed to assess the potential of digital biomarkers for personalized classification of PPD, we sought to further examine each RF model’s performance via a confusion matrix. Thus, the average sensitivity, specificity, precision, recall, and *F*_1_-score were calculated across all individual models, where the results for the PPD class were 0.79, 0.95, 0.84, 0.79, and 0.81, respectively (Figure S1 in Multimedia Appendix 2). The same metrics for the prepregnancy, pregnancy, and postpartum periods were also calculated (Figure S1 in Multimedia Appendix 2).

To ensure the widespread applicability of these algorithms to a diverse range of women, we did not exclude individuals with a history of depression either before or during pregnancy. Therefore, we sought to determine whether having depression before or during pregnancy impacted individual model performance, specifically for recognizing the PPD class. To answer this question, we computed the average sensitivity, specificity, precision, recall, and *F*_1_-score within the group of women experiencing PPD categorized based on their depression history: (1) no previous history of depression, (2) history before pregnancy, (3) history during pregnancy, or (4) history both before and during pregnancy. Notably, the findings revealed no statistically significant variations in any of these metrics between women with a history of depression during the prepregnancy or pregnancy periods and those without such a history (Figure S2 in Multimedia Appendix 2). Promisingly, this suggests the potential for a forthcoming technology focused on detecting PPD through digital biomarkers to be relevant for women with or without a previous history of depression before or during pregnancy.

### Individualized ML Models for PPD Recognition Were Specific

To validate our approach of using digital biomarkers in individualized ML models for PPD detection, we aimed to test our strategy in a cohort of women who had given birth but did not experience PPD. We chose women without PPD as a control group for validation because they experienced the same 3 phases of pregnancy (prepregnancy period, pregnancy, and postpartum period) as women in the PPD cohort with the exception of PPD. Given that women without PPD did not have a distinct PPD-specific period as observed in the PPD cohort, we introduced a fourth time segment in the non-PPD cohort (the PPD-equivalent period). Following the same ML pipeline as for the PPD cohort, individualized RF models were built for women in the non-PPD cohort. If our conjecture held, we anticipated observing elevated model metrics during the prepregnancy and pregnancy periods followed by diminished performance in the postpartum and PPD-equivalent time segments. This expectation arose from the idea that digital biomarkers remain unaltered during the postpartum and PPD-equivalent periods, resulting in the model’s inability to differentiate between them.

**Figure 4:**
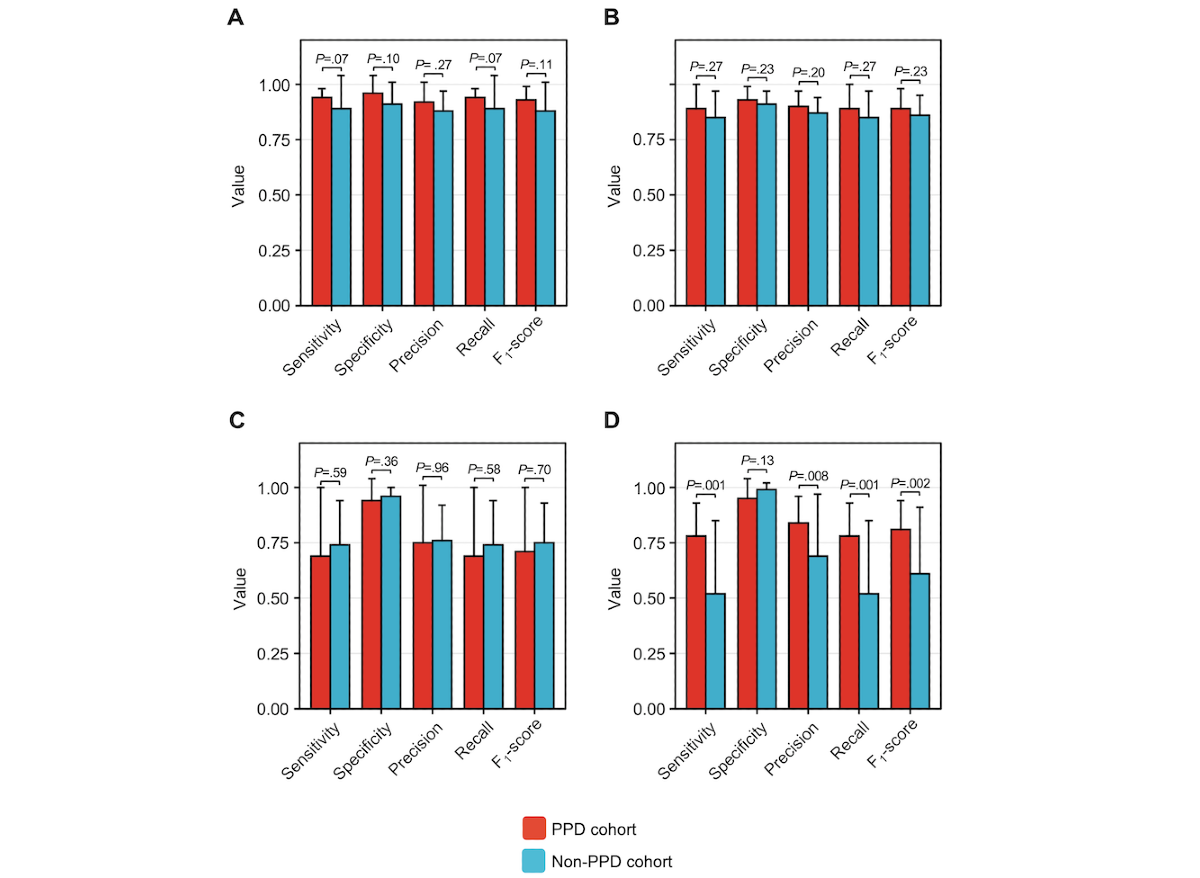
Individualized machine learning models for postpartum depression (PPD) recognition outperformed those in women without PPD detecting the PPD-equivalent period. The sensitivity, specificity, precision, recall, and F1-score were calculated across individual random forest models for women in the PPD and non-PPD cohorts for the prepregnancy (A), pregnancy (B), postpartum (C), and PPD or PPD-equivalent periods (D). Individualized model performance was not significantly different regarding sensitivity, specificity, precision, recall, and F1-score for predicting the prepregnancy, pregnancy, or postpartum periods between women in the PPD and non-PPD cohorts. Individualized model performance was reduced for sensitivity, precision, recall, and F1-score, whereas specificity did not differ between the PPD and non-PPD cohorts. Data are expressed as mean and SD.

In line with our hypothesis, the sensitivity, specificity, precision, recall, and *F*_1_-scores substantiated that ML models effectively identified the prepregnancy (0.89, 0.91, 0.88, 0.89, and 0.88, respectively) and pregnancy (0.85, 0.91, 0.87, 0.85, and 0.86, respectively) time intervals through digital biomarkers (Table 3). When compared to model performance in the prepregnancy and pregnancy periods, there was no significant reduction in model performance during the postpartum period (0.74, 0.96, 0.76, 0.74, and 0.75, respectively); however, a noticeable decline in performance was observed during the PPD-equivalent period (0.52, 0.99, 0.69, 0.52, and 0.61, respectively; Table 3). To further assess potential variations in the classification performance between the PPD and PPD-equivalent periods, we carried out a *t* test comparing the average sensitivity, specificity, precision, recall, and *F*_1_-score between the PPD and non-PPD cohorts for these periods. The findings indicated a statistically significant decrease in sensitivity, precision, recall, and *F*_1_-score when predicting the PPD-equivalent period in the non-PPD cohort as opposed to predicting the PPD period in the PPD cohort (Figure 4). On the other hand, specificity remained largely unchanged (Figure 4). The decrease in performance among individualized ML models in the PPD-equivalent period implies that the models were unable to accurately classify the PPD-equivalent period, which was expected as there was no actual distinction between the postpartum and PPD-equivalent periods for these women. Collectively, these outcomes helped demonstrate the specificity of our approach in identifying PPD, reinforcing the agreement that personalized models using digital biomarkers can indeed effectively recognize PPD.

**Table 3:** Machine learning (ML) models did not accurately detect the postpartum depression (PPD)–equivalent period in women without PPD.

Time period and metric			Value, mean (SD)
**Prepregnancy period**			
	Sensitivity	0.89 (0.15)	
	Specificity	0.91 (0.10)	
	Precision	0.88 (0.09)	
	Recall	0.89 (0.15)	
	*F*_1_-score	0.88 (0.13)	
**Pregnancy period**			
	Sensitivity	0.85 (0.12)	
	Specificity	0.91 (0.06)	
	Precision	0.87 (0.07)	
	Recall	0.85 (0.12)	
	*F*_1_-score	0.86 (0.09)	
**Postpartum period**			
	Sensitivity	0.74 (0.20)	
	Specificity	0.96 (0.04)	
	Precision	0.76 (0.16)	
	Recall	0.74 (0.20)	
	*F*_1_-score	0.75 (0.18)	
**PPD-equivalent period**			
	Sensitivity	0.52 (0.33)	
	Specificity	0.99 (0.03)	
	Precision	0.69 (0.28)	
	Recall	0.52 (0.33)	
	*F*_1_-score	0.61 (0.30)	

### Calories BMR Was the Most Predictive Digital Biomarker of PPD

To elucidate which digital biomarkers were most predictive of the PPD class, we performed SHAP to explain individual predictions for each digital biomarker across all RF intraindividual models [59]. Features were sorted based on their predictive value for the PPD class within each individual model, and subsequently, the occurrences of each digital biomarker ranking in the top 5 across all intraindividual models were tallied, where an example beeswarm plot for one woman is shown in Figure S3 in Multimedia Appendix 2. This process aimed to identify whether any digital biomarkers consistently played a crucial role in predicting the PPD class. The results showed that the 5 features most frequently ranked in the top 5 were calories BMR, average HR, quartile 1 HR, lightly active minutes, and minimum HR (Table 4). Interestingly, calories BMR ranked in the top 5 features predictive of the PPD class in 95% to 100% of the models and was the number 1 rated digital biomarker in 80% to 85% of the individualized models (Table 4).

To add a layer of robustness to our approach assessing which features were most predictive of the PPD class, the variable importance of each digital biomarker was also calculated using a permutation approach [60]. Consistent with our findings obtained using SHAP, the top 5 digital biomarkers for the PPD class were calories BMR, average HR, quartile 1 HR, minimum HR, and lightly active minutes (Table 4). Calories BMR again ranked in the top 5 digital biomarkers predictive of the PPD class 95% to 100% of the time and ranked number one 95% to 100% of the time (Table 4).

**Figure 5:**
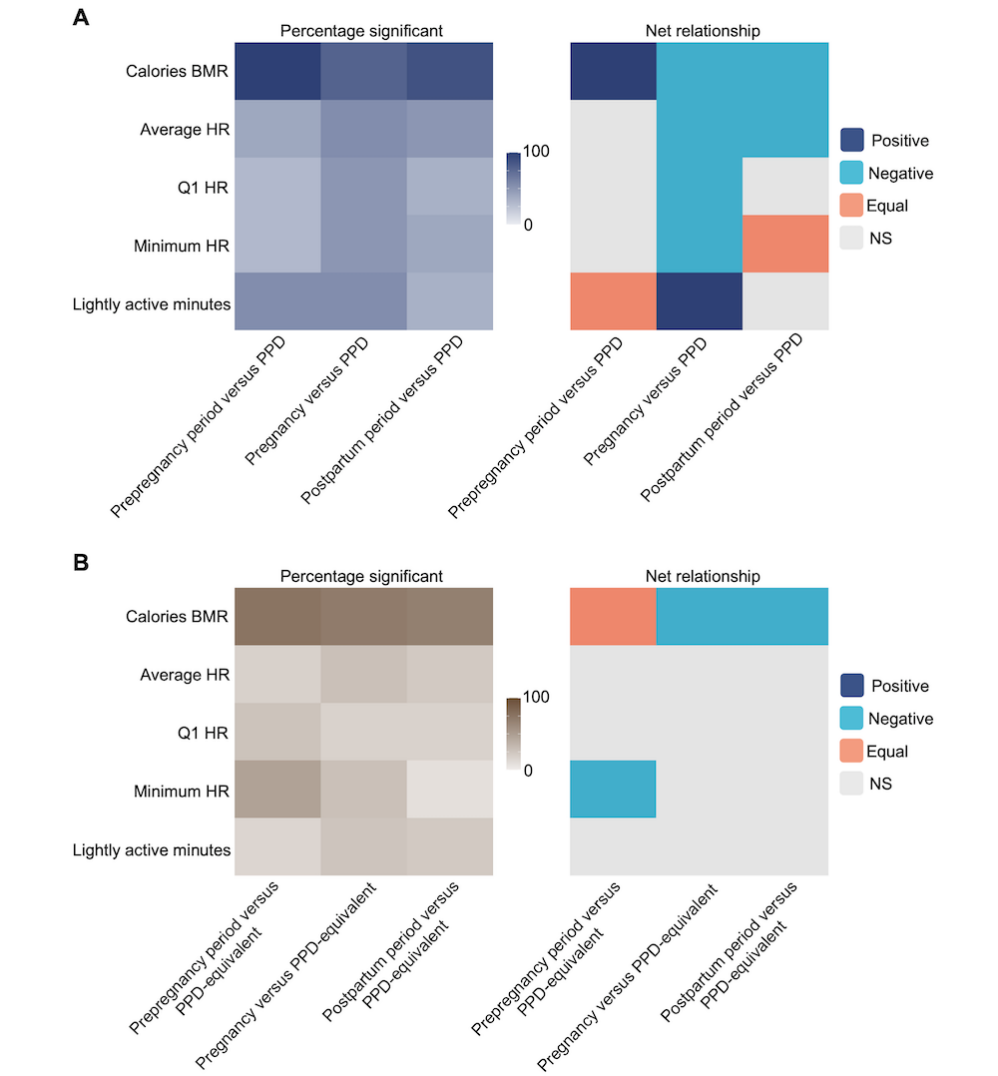
The direction of digital biomarkers in machine learning models for postpartum depression (PPD) classification was heterogeneous. (A) The percentage of women in the PPD cohort with a significant Pearson correlation (left) and the net relationship (right) for the top 5 overall ranked digital biomarkers for PPD classification. (B) The percentage of women in the non-PPD cohort with a significant Pearson correlation (left) and the net relationship (right) for the top 5 overall ranked digital biomarkers for PPD-equivalent classification. The proportion of women showing a significant Pearson correlation coefficient between Shapley additive explanations (SHAP) values and digital biomarkers varied in both the PPD and non-PPD cohorts. The x-axis illustrates the comparison of 2 periods, with the first period as the reference, whereas the shading indicates the percentage (0%-100%) of women showing a significant relationship (A) or the net relationship among those with a significant relationship (B). SHAP dependence plots were generated for each woman on an individual basis. For instance, the upper left tile in A presenting prepregnancy versus PPD and Calories BMR indicates the percentage of women who showed a significant correlation on SHAP dependence plots of calories burned during the basal metabolic rate (calories BMR) between the prepregnancy and PPD periods. In B, the upper left tile of the heat map for prepregnancy versus PPD and Calories BMR illustrates that most women in the PPD cohort showed a positive relationship (elevated SHAP values with increases in calories BMR, meaning that a higher level of calories BMR was more predictive of PPD than in the prepregnancy period) among women with a significant correlation, as shown in A. Among those showing a significant relationship in SHAP dependence plots during the prepregnancy versus PPD (and prepregnancy versus PPD-equivalent) periods, the correlation pattern for SHAP values and calories BMR differed—most women exhibited a positive correlation in the PPD cohort, whereas there was no uniform pattern among women in the non-PPD cohort. Among women in the pregnancy versus PPD and postpartum versus PPD (and PPD-equivalent) periods, most demonstrated a negative relationship between SHAP values and calories BMR in both the PPD and non-PPD cohorts. HR: heart rate; NS: not significant; Q1: quartile 1.

Because of the intriguing observation that calories BMR was highly predictive of PPD across all models, we sought to better understand its relationship with the PPD class in our models using SHAP dependence plots to visualize and calculate the Pearson correlation coefficient between the PPD period and the prepregnancy, pregnancy, or postpartum periods (see Figure S4A-C in Multimedia Appendix 2 for example plots from individual women). Across all individual SHAP dependence plots of calories BMR filtered in the prepregnancy versus PPD periods, our initial observation revealed that 95% to 100% of women exhibited a significant Pearson correlation coefficient (Figure 5A). Of these women, 60% to 65% showed a positive relationship, indicating an elevated level of calories BMR during the PPD period relative to the prepregnancy period (Figure 5A). Compared to the prepregnancy versus PPD period, it was observed that 75% to 80% and 85% to 90% of individualized SHAP dependence plots of calories BMR during the pregnancy versus PPD and postpartum versus PPD periods exhibited a significant Pearson correlation coefficient, respectively (Figure 5A). Of those, 60% to 65% and 85% to 90% of women during the pregnancy versus PPD and postpartum versus PPD periods demonstrated a negative relationship, respectively, suggesting that a decrease in calories BMR relative to the pregnancy and postpartum periods was predictive of PPD (Figure 5A). SHAP dependence plots were also generated for individualized models of the other top 4 digital biomarkers predictive of PPD (average HR, quartile 1 HR, minimum HR, and lightly active minutes) in the prepregnancy versus PPD, pregnancy versus PPD, and postpartum versus PPD periods (Figure 5A). Notably, during the prepregnancy versus PPD periods, half of the women exhibited a positive relationship in plots of lightly active minutes, indicating an increase in lightly active minutes associated with PPD in those models (Figure 5A). To examine the rise in lightly active minutes relative to other digital biomarkers of physical activity (sedentary minutes, fairly active minutes, and very active minutes), we calculated the ratio of the number of lightly active minutes to each of the 3 other digital biomarkers of physical activity across all individuals. In this case, we observed that the average ratios of lightly active minutes to sedentary minutes, fairly active minutes, and very active minutes were 0.35 (SD 0.49), 17.7 (SD 4.92), and 21.72 (SD 5.11), respectively (Figure S5 in Multimedia Appendix 2).

**Table 4:** The variable importance rankings demonstrated that calories burned during the basal metabolic rate (calories BMR) were the most predictive digital biomarker of the postpartum depression (PPD) class.

Method and digital biomarker			Percentage ranked top 5		Percentage ranked number 1
**SHAP^a^**					
	Calories BMR	100		85	
	Average HR^b^	40		0	
	Quartile 1 HR	40		0	
	Lightly active minutes	35		10	
	Minimum HR	35		0	
	Sedentary minutes	0		10	
	Sum of steps	0		10	
**Permutation**					
	Calories BMR	100		100	
	Average HR	65		0	
	Quartile 1 HR	60		0	
	Minimum HR	50		0	
	Lightly active minutes	40		0	

^a^SHAP: Shapley additive explanations.

^b^HR: heart rate.

For a more comprehensive evaluation of the connection between calories BMR and PPD, we also crafted SHAP dependence plots from individualized ML models for women without PPD. When first assessing the number of women with a significant correlation in SHAP dependence plots of the prepregnancy versus PPD-equivalent, pregnancy versus PPD-equivalent, and postpartum versus PPD-equivalent periods, the results showed that 75% to 80%, 70% to 75%, and 65% to 70% of women had a significant relationship, respectively (Figure 5B). Of those, there was an equal number of women with a positive and negative relationship in the prepregnancy versus PPD-equivalent periods compared to the PPD cohort, where most women (60%-65%) exhibited a positive relationship (Figure 5B). This implies that, among women in the PPD cohort, an escalation in calories BMR corresponds to a higher likelihood of PPD when compared to the prepregnancy period (Figures 5A and 5B). On the other hand, in the non-PPD cohort, there was no uniform pattern of association between calories BMR during the prepregnancy and the PPD-equivalent periods across all women, highlighting the distinctive nature of our observation. During the pregnancy versus PPD-equivalent and postpartum versus PPD-equivalent time frames, 80% to 85% and 75% to 80% of women, respectively, exhibited a significant correlation in SHAP dependence plots between calories BMR and Shapley values (Figure 5B). As anticipated, this follows a similar pattern to women in the PPD cohort (Figure 5A). These findings implied that a reduction in calories BMR compared to the pregnancy or postpartum periods is linked to the PPD (or PPD-equivalent) periods (Figures 5A and 5B).

We further investigated (1) health care–seeking behavior among women in the PPD and non-PPD cohorts; (2) the reliability of self-reported weight (as calories BMR are calculated based on age, sex, height, and weight); (3) the weight difference across each of the 4 periods (prepregnancy period, pregnancy, postpartum period, and PPD [or PPD equivalent]) between women in the PPD and non-PPD cohorts; (4) the relationship among weight, calories BMR, and PPD; and (5) weight retention between women in the PPD and non-PPD cohorts (see more details in Tables S5-S11 in Multimedia Appendix 2).

To showcase the effectiveness of our approach using individualized ML models for PPD detection, we constructed an ML model using conventional techniques. In this endeavor, we harnessed the PPD and PPD-equivalent periods of the PPD and non-PPD cohorts, respectively, enabling an assessment of our individualized approach compared to conventional methods using a binomial model for the classification of individuals with or without PPD. By evaluating model outcomes through metrics such as sensitivity, specificity, precision, recall, and *F*_1_-score, we found that the average performance of the individualized model surpassed that of the cohort-based strategy (Table 5). Specifically, in the individualized approach, we observed sensitivity, specificity, precision, recall, and *F*_1_-score values of 0.78, 0.95, 0.84, 0.78, and 0.81, respectively, in contrast to 0.54, 0.55, 0.49, 0.54, and 0.52, respectively, for the cohort-based approach (Table 5).

**Table 5:** Individualized machine learning (ML) models outperformed a cohort-based model for postpartum depression (PPD) recognition.

Method and digital biomarker			Value
**Sensitivity**			
	Cohort-based model^a^	0.54	
	Individualized models, mean (SD)	0.78 (0.15)	
**Specificity**			
	Cohort-based model	0.55	
	Individualized models, mean (SD)	0.95 (0.09)	
**Precision**			
	Cohort-based model	0.49	
	Individualized models, mean (SD)	0.84 (0.12)	
**Recall**			
	Cohort-based model	0.54	
	Individualized models, mean (SD)	0.78 (0.15)	
***F*_1_-score**			
	Cohort-based model	0.52	
	Individualized models, mean (SD)	0.81 (0.13)	

^a^Since the cohort-based ML approach is only to generate 1 model, there is no mean or SD.

## Discussion

### Principal Findings

In this study, our multifaceted analysis demonstrated that (1) digital biomarkers differed among the prepregnancy, pregnancy, and postpregnancy periods (up to 2 years before pregnancy, pregnancy, postpartum period, and PPD; Figure 3 and Tables S3 and S4 in Multimedia Appendix 2); (2) personalized N-of-1 ML models using digital biomarkers from consumer-grade wearables were able to classify PPD and other periods of pregnancy (Table 2 and Figure S1 in Multimedia Appendix 2); (3) a history of depression before or during pregnancy did not impact individualized ML model performance for PPD recognition (Figure S2 in Multimedia Appendix 2); (4) calories BMR, average HR, quartile 1 HR, lightly active minutes, and minimum HR were the most influential digital biomarkers in predicting the PPD period across all individualized models (Table 4); and (5) individualized ML models for PPD recognition outperformed the traditional cohort-based model approach (Table 5). The results presented in this paper provide a new opportunity for the potential to leverage passively collected digital biomarkers from consumer-grade wearables to facilitate early detection of PPD.

To the best of our knowledge, this is the first study proposing that individualized ML models using passively collected digital biomarkers from consumer-grade wearables can recognize PPD. Moreover, this study is also unique due to (1) integrating EHR and wearable data sources, (2) using longitudinal and continuous wearable data across multiple periods of pregnancy for ML methods, and (3) using individualized ML models for PPD recognition. PPD is most commonly diagnosed using the EPDS, which suffers from the following limitations: (1) postpartum women must attend follow-up visits assessed by care providers for PPD screening, where the rate of postpartum visits is highly variable; (2) using the EPDS only captures the mental health of a woman at a single point in time; and (3) the EPDS uses self-reported symptoms, which may not be representative of a patient’s actual mental health status [61-63]. For these reasons, our approach using passively monitored digital biomarkers from consumer wearable technology may serve as an effective tool for facilitating the detection of PPD in an individualized fashion, especially in nonclinical settings.

Because of the variation in digital biomarkers detected among women across different periods, our limited sample size, and the availability of continuous intraindividual data, our study was geared toward an individualized analytic approach (Table S2 in Multimedia Appendix 2). The observed variability across individuals is consistent with previous studies that have emphasized the heterogeneous nature of depression prompting individualized methodologies [16,54,64-66]. Moreover, ITSA and Tukey HSD results revealed that digital biomarkers were significantly altered among periods within each woman (Figure 3 and Tables S3 and S4 in Multimedia Appendix 2). Overall, there were numerous individual-level alterations, which can be explained by the considerable heterogeneity in depressive symptoms [54]. Collectively, these data suggest that digital biomarkers were significantly different across periods within each person, leading us to believe that individualized ML models would be able to accurately discriminate between PPD and other periods of pregnancy.

Our study also highlights the strength of using individualized N-of-1 ML models using digital biomarkers for identifying PPD. Our findings underscored the models’ ability to differentiate between distinct pregnancy phases—namely, prepregnancy, pregnancy, postpartum, and PPD periods (Table 2 and Figure S1 in Multimedia Appendix 2). Notably, our approach’s validity was confirmed by the noticeable decrease in model performance during the PPD-equivalent period for the non-PPD cohort compared to the PPD period for the PPD cohort (Table 3 and Figure 4). This demonstrated the distinct behavioral shifts that are observed during the onset of PPD, effectively captured by digital biomarkers [2]. Furthermore, our results did not indicate a significant variation in individualized model performance across the 4 pregnancy periods among women with a history of depression before or during pregnancy (Figure S2 in Multimedia Appendix 2). This accentuated the robust capability of individualized models to differentiate among periods based on the distinct behavioral characteristics and metabolic shifts linked to PPD after pregnancy as opposed to the behavior changes exhibited by each woman before or during pregnancy. This suggests that forthcoming technology centered on detecting PPD through digital biomarkers could have relevance for both individuals with and without a preexisting history of depression before or during pregnancy. Future studies should be conducted in a prospective framework to validate our individualized methodology. We also want to emphasize in future studies the importance of ML algorithms minimizing false positives for PPD detection to prevent unnecessary interventions [67].

Another crucial finding of our study was that the vital digital biomarkers for PPD classification were calories BMR, average HR, quartile 1 HR, minimum HR, and lightly active minutes, where calories BMR was the most predictive feature (Table 4). Therefore, we constructed SHAP dependence plots to enhance our understanding of the relationship between calories BMR and PPD. Plots for the prepregnancy versus PPD periods suggested that an elevated level of calories BMR is predictive of PPD, which is indicative of weight gain in these women (Figure 5A) [2,68]. In plots for the pregnancy versus PPD and postpartum versus PPD periods, the relationship between Shapley and actual values of calories BMR flipped, signifying that an increased number of calories BMR was inversely associated with PPD (Figure 5A). The negative relationship in the context of pregnancy versus PPD can likely be explained by the metabolic changes during pregnancy, resulting in an increased basal metabolic rate [69]. In the context of the postpartum versus PPD periods, we speculate that the negative relationship is because the median duration between the delivery date and PPD diagnosis is 83 days, when patients may not have fully returned to their prepregnancy physiological or behavioral patterns, which can take up to 6 months [70]. As a result, the relationship between Shapley values and actual values of calories BMR may reflect this transitional period and the ongoing postpartum changes experienced by women.

On the other hand, for the prepregnancy versus PPD-equivalent periods for women in the non-PPD cohort, SHAP dependence plots failed to unveil a uniform connection between calories BMR and the PPD-equivalent period, likely due to physiological distinctions, lifestyle changes during pregnancy, and random dissimilarities among women [57,71-73]. However, the comparison of SHAP dependence plots across the pregnancy versus PPD-equivalent and postpartum versus PPD-equivalent periods for women in the non-PPD cohort exhibited a consistent negative correlation, similar to what was observed in the PPD cohort (Figures 5A and 5B). This trend is likely a result of the common occurrence of an increased basal metabolic rate during pregnancy [69]. In the context of the postpartum versus PPD-equivalent periods, our use of an index date set at 83 days after delivery—the median number of days after delivery for PPD diagnosis in the PPD cohort—implies that women likely have not fully returned to their prepregnancy physiological baseline [70]. This aligns with the parallel observation seen during the pregnancy versus PPD-equivalent periods, reaffirming the persisting metabolic effect postpartum.

Calories BMR are calculated using a combination of age, gender, height, and weight. Hence, considering the relative stability of age, gender, and height throughout the 4 phases of pregnancy (prepregnancy period, pregnancy, postpartum period, and PPD), alterations in calories BMR are likely indicative of weight changes [74]. Calories BMR likely rely on self-reported height and weight measurements as most individuals do not have routine access to gold-standard measurements. Previous research has indicated that self-reported weight is generally reliable and accurate, where BMI was correctly determined for 91% of pregnancies using self-reported weight; however, accuracy varied between 70% in women who are underweight and 98% in women who are overweight [75]. In our study, we found no significant difference in self-reported and gold-standard weight measurements (Table S6 in Multimedia Appendix 2). Hence, it is important to acknowledge that an ideal approach would involve a combination of gold-standard weight measurements for accuracy and self-reported weight measurements for feasibility and accessibility to longitudinal data in analyses related to PPD.

Given the known positive relationship between calories BMR and weight, our analyses sought to further examine this relationship in the context of women with PPD. To do so, we leveraged the results from SHAP dependence plots, where we found that the 60% to 65% of women with a positive relationship between calories BMR and PPD relative to the prepregnancy period (ie, a higher value of calories BMR during PPD compared to the prepregnancy period) experienced weight gain (Table S9 in Multimedia Appendix 2). Similarly, the 85% to 90% of women who showed a negative relationship between calories BMR and PPD relative to the postpartum period (ie, a lower value of calories BMR during PPD compared to the postpartum period) showed weight loss (Table S10 in Multimedia Appendix 2). It is worth noting that previous research has suggested that some women experience weight gain during PPD, whereas others experience weight loss. For instance, it has been shown that women with PPD may experience weight gain attributed to emotional overeating [76,77]. Conversely, other studies propose that women grappling with PPD might experience weight loss resulting from skipped meals and overwhelming anxiety [78]. Consistent with the individualized framework presented in our study, we posit that it may be crucial to monitor changes in weight (or calories BMR). For example, although most women in our study exhibited an increase in calories BMR during the PPD period compared to the prepregnancy period, a percentage of women experienced a significant decrease in calories BMR during PPD relative to the prepregnancy period. When assessing the relationship between weight in the prepregnancy and PPD periods in these women, we found that these women did experience weight loss during the PPD period relative to the prepregnancy period. Therefore, we suggest that changes in body weight (or calories BMR), whether positive or negative, could potentially serve as more informative indicators of PPD. Considering the diverse manifestations of depression—some individuals may gain weight, whereas others may lose weight—we advocate for future studies to investigate these changes on an individual level.

Considering previous research indicating that women with PPD encounter challenges in reverting to prepregnancy weight compared to those without PPD, we aimed to assess this phenomenon in our cohorts. Strikingly, our findings did not detect a significant difference in the time taken for women to reach their prepregnancy weight between the PPD and non-PPD cohorts (Table S11 in Multimedia Appendix 2). Due to the association between calories BMR and weight, we aimed to validate this observation using calories BMR data. Interestingly, we observed a consistent pattern of no significant difference in the time taken to return to prepregnancy calories BMR between the PPD and non-PPD cohorts (Table S11 in Multimedia Appendix 2). It is posited that the lack of difference may be because women in the PPD cohort started at a higher average weight during the prepregnancy period compared to those without PPD (Table S8 in Multimedia Appendix 2). We also suspect that the discrepancy in the results between the number of days to return to prepregnancy weight detected via weight measurements and calories BMR was a product of the limited availability of weight data.

In the PPD cohort, the SHAP dependence plots for average HR, quartile 1 HR, and minimum HR during the pregnancy versus PPD periods also demonstrated a negative relationship, indicating that higher values of these digital biomarkers are inversely associated with PPD (Figure 5A). This relationship may be ascribed to the elevated HR commonly observed during pregnancy, which is a physiological response resulting from vascular remodeling for promoting augmented blood flow to the uterus [79-81]. In addition, there was a positive correlation between the increase in lightly active minutes and the occurrence of PPD in the pregnancy versus PPD periods, which may be explained by an inverse relationship between lightly active minutes and fairly active minutes or very active minutes (Figure S5 in Multimedia Appendix 2). Specifically, a higher number of lightly active minutes is concomitant with a decrease in the amount of time spent in fairly active and very active physical activities, aligning with the well-established understanding that reductions in overall physical activity can contribute to an increase in depressive symptoms [82]. In contrast, among women without PPD, a notable correlation was found solely in the prepregnancy versus PPD-equivalent periods concerning minimum HR, where an elevation in minimum HR was linked to the PPD-equivalent period (Figure 5B). Although a subset of women demonstrated a significant correlation in SHAP dependence plots concerning digital biomarkers of average HR, quartile 1 HR, or lightly active minutes across the prepregnancy versus PPD-equivalent, pregnancy versus PPD-equivalent, or postpartum versus PPD-equivalent periods, the overall proportion of women exhibiting such patterns was insufficient to draw definitive conclusions regarding the relationship between digital biomarkers during the prepregnancy, pregnancy, or postpartum periods and the PPD-equivalent period (Figure 5B). We postulate that the contrasting patterns of digital biomarkers between women in the PPD and non-PPD cohorts imply potential differences in these biomarkers for women who eventually experience PPD. Therefore, future studies of great interest may seek to develop ML models during the prepregnancy or pregnancy periods to predict a woman’s risk of future PPD onset. These models would allow for the prediction of PPD risk in advance.

### Comparison With Prior Work

In general, previous investigations have adhered to conventional ML strategies revolving around the development of a solitary model. In this paradigm, a model is constructed using an extensive patient data set encompassing individuals exhibiting either continuous outcomes (for regression-based models) or categorical outcomes. Subsequently, when a new patient is introduced, the model generates predictions for the patient based on their data and the pre-established model [83]. While this approach carries advantages, it is beset by two primary limitations: (1) reliance on an ample sample size and (2) neglect to accommodate the diverse and heterogeneous spectrum of depressive symptoms [16]. Hence, a captivating domain of exploration has honed in on crafting intraindividual ML models. This advancement tackles the constraints of conventional approaches in 2 ways: first, it sidesteps the need for an extensive sample size given that the model is tailored to a single patient’s data, and second, it conscientiously acknowledges the heterogeneous spectrum of depressive symptoms through a focused evaluation of the unique behaviors exhibited by that specific patient.

The use of individualized models may serve as a superior preference compared to those formulated using cohort-based methodologies. For instance, a cross-sectional study using traditional ML models from Fitbit data from healthy adults to predict depression severity only showed a moderate area under the receiver operating characteristic curve range of 0.51 to 0.66. Moreover, while the results demonstrated commendable specificity (0.98-1), sensitivity exhibited marked inadequacy (0.03-0.13) [19]. Another study aimed to investigate the potential of ML models using digital biomarkers in distinguishing between patients with unipolar and bipolar depression against healthy controls. However, the most successful model exhibited an accuracy rate of 0.73 (73%) and a κ value of 0.44, which does not indicate a notably high-performing model [84,85]. Additional investigations have also been conducted within a cohort-based framework; nevertheless, these studies grapple with a noteworthy drawback—they incorporated patient mood as a predictive feature in their models. Considering that these studies aimed to predict the severity of depression, it is unsurprising that these models exhibited heightened performance levels [13,86].

To effectively underscore the viability of personalized ML models over cohort-based methods, our study directly juxtaposed the performance of both approaches (individualized vs cohort-based ML models) side by side. Notably, our findings vividly showcased the superior performance achieved through the personalized methodology for the PPD class in comparison to conventional techniques with a cohort-based model leveraging digital biomarkers from Fitbit for PPD detection (Table 5). This outcome accentuated that individualized models present an encouraging avenue for crafting ML models aimed at identifying mood disorders.

### Limitations

Although this study provides a strong foundation for using digital biomarkers to classify PPD, it is not without limitations. First, this study faced constraints due to the restricted number of patients available, which hindered the implementation of conventional ML techniques. However, due to the limited sample size, we opted for an individualized approach, which not only addressed the small sample size but also provided a means to accommodate the inherent variability among individuals [54]. Second, the process of phenotyping patients with PPD relied on a PPD diagnosis or medication use, which could potentially lack specificity in diagnostic codes and miss undiagnosed cases. Third, our approach assumed a standard pregnancy length of 9 months, which may not always align with individual variations. Fourth, there are several layers of confounding that occur during the different phases of pregnancy that may indirectly influence digital biomarkers and ML models, especially as it relates to PPD classification, such as (1) significant hormonal changes that impact physical and mental states; (2) metabolic changes that occur as a result of pregnancy; (3) increased levels of stress during pregnancy and the postpartum period; (4) modifications to one’s lifestyle, such as food consumption during pregnancy and the postpartum period; and (5) alterations in physical activity during the postpartum period as a result of birthing complications [87-91]. This could create a risk of model overfitting on general postpartum features compared to those that are specific to the PPD period. In general, to avoid overfitting of the training data, our method used 3 repetitions of 10-fold cross-validation, a strategy known to reduce overfitting compared to a conventional train-test split [92]. Fifth, this study excluded patients with chronic conditions to mitigate the potential influence of those conditions on digital biomarkers. Sixth, sleep data were absent in the AoURP data set at the time of this analysis using Registered Tier v6 although they might also hold predictive value for PPD. Seventh, there is a possibility of false negatives in the non-PPD cohort given the 1 in 7 prevalence of PPD. We attempted to identify women in the non-PPD cohort with undiagnosed PPD by re-evaluating individual model performance for the PPD-equivalent class, where we counted approximately 10 women in the non-PPD cohort who exhibited results with elevated model performance (sensitivity≥0.78 [the average performance of the PPD class for women in the PPD cohort]). However, estimating the prevalence of undiagnosed PPD is difficult in itself; estimating undiagnosed PPD in EHR data is additionally challenging. We suggest that these exploratory findings require future model development, including true negatives (ie, women who are definitely not experiencing PPD) and false negatives for validation. Eighth, there may be delays in PPD diagnosis due to health care–seeking behavior. However, we observed no significant difference in health care use during the postpartum period between the PPD and non-PPD cohorts along with similar adherence to American College of Obstetricians and Gynecologists postpartum visit guidelines in the PPD and non-PPD cohorts. We posit that delays in diagnosis due to health care–seeking behavior might be minimal (Table S5 in Multimedia Appendix 2). These data suggest that women in the PPD cohort exhibited a fairly normal rate of health care use, which should minimize delay in diagnosing PPD. Furthermore, it may be beneficial for subsequent analyses to account for features such as seasonal variation that may also indirectly influence behavior (which is not possible using the Registered Tier of AoURP) in addition to testing other ML algorithms, such as Extreme Gradient Boosting [93,94].

### Conclusions

Overall, the findings of this study suggest that it is feasible to characterize PPD in addition to other periods of pregnancy using passively collected digital biomarkers from consumer-grade wearables. The development of individualized models allows for a personalized approach to capture behavioral differences in the form of digital biomarkers. This research lays a robust foundation for forthcoming applications aimed at enhancing the early detection of PPD, a condition that is often underdiagnosed and undertreated. Moreover, on a broader scale, it indicates the exciting potential for intraindividual ML models to be extended to various health conditions.

## Appendix

Multimedia Appendix 1 Observational Medical Outcomes Partnership concept IDs used in computational phenotyping of postpartum depression (PPD) and non-PPD cohorts.

Multimedia Appendix 2 Supplementary results, tables, and figures.

## Abbreviations

AoURP: *All of Us* Research Program
Calories BMR: calories burned during the basal metabolic rate
EHR: electronic health record
EPDS: Edinburgh Postnatal Depression Scale
GLM: generalized linear model
HR: heart rate
HSD: honest significant difference
ITSA: interrupted time-series analysis
KNN: k-nearest neighbor
mAUC: multiclass area under the receiver operating characteristic curve
ML: machine learning
PPD: postpartum depression
RF: random forest
SHAP: Shapley additive explanations
SVM: support vector machine

## Data Availability

The data sets generated during and analyzed during this study are not publicly available due to patient privacy restrictions and guidelines established by the All of Us Research Program that require individuals with access to the platform complete the requisite training, but they are available from the corresponding author on reasonable request.

## References

[1] Gaynes BN, Gavin N, Meltzer-Brody S, Lohr KN, Swinson T, Gartlehner G, Brody S, Miller WC (2005). Perinatal depression: prevalence, screening accuracy, and screening outcomes. Evid Rep Technol Assess (Summ).

[2] Pearlstein T, Howard M, Salisbury A, Zlotnick C (2009). Postpartum depression. Am J Obstet Gynecol.

[3] Luca DL, Margiotta C, Staatz C, Garlow E, Christensen A, Zivin K (2020). Financial toll of untreated perinatal mood and anxiety disorders among 2017 births in the United States. Am J Public Health.

[4] Kavanaugh M, Halterman JS, Montes G, Epstein M, Hightower AD, Weitzman M (2006). Maternal depressive symptoms are adversely associated with prevention practices and parenting behaviors for preschool children. Ambul Pediatr.

[5] Duan Z, Wang Y, Jiang P, Wilson A, Guo Y, Lv Y, Yang X, Yu R, Wang S, Wu Z, Xia M, Wang G, Tao Y, Xiaohong L, Ma L, Shen H, Sun J, Deng W, Yang Y, Chen R (2020). Postpartum depression in mothers and fathers: a structural equation model. BMC Pregnancy Childbirth.

[6] Hutchens BF, Kearney J (2020). Risk factors for postpartum depression: an umbrella review. J Midwifery Womens Health.

[7] Sit DK, Wisner KL (2009). Identification of postpartum depression. Clin Obstet Gynecol.

[8] Evins GG, Theofrastous JP (1997). Postpartum depression: a review of postpartum screening. Prim Care Update Ob Gyns.

[9] Fitelson E, Kim S, Baker AS, Leight K (2010). Treatment of postpartum depression: clinical, psychological and pharmacological options. Int J Womens Health.

[10] Mobile fact sheet. Pew Research Center.

[11] Avram R, Tison G, Kuhar P, Marcus G, Pletcher M, Olgin JE, Aschbacher K (2019). Predicting diabetes from photoplethysmography using deep learning. J Am Coll Cardiol.

[12] Mishra T, Wang M, Metwally AA, Bogu GK, Brooks AW, Bahmani A, Alavi A, Celli A, Higgs E, Dagan-Rosenfeld O, Fay B, Kirkpatrick S, Kellogg R, Gibson M, Wang T, Hunting EM, Mamic P, Ganz AB, Rolnik B, Li X, Snyder MP (2020). Pre-symptomatic detection of COVID-19 from smartwatch data. Nat Biomed Eng.

[13] Narziev N, Goh H, Toshnazarov K, Lee SA, Chung KM, Noh Y (2020). STDD: short-term depression detection with passive sensing. Sensors (Basel).

[14] Steinhubl SR, Mehta RR, Ebner GS, Ballesteros MM, Waalen J, Steinberg G, Van Crocker P, Felicione E, Carter CT, Edmonds S, Honcz JP, Miralles GD, Talantov D, Sarich TC, Topol EJ (2016). Rationale and design of a home-based trial using wearable sensors to detect asymptomatic atrial fibrillation in a targeted population: the mHealth Screening To Prevent Strokes (mSToPS) trial. Am Heart J.

[15] Pedrelli P, Fedor S, Ghandeharioun A, Howe E, Ionescu DF, Bhathena D, Fisher LB, Cusin C, Nyer M, Yeung A, Sangermano L, Mischoulon D, Alpert JE, Picard RW (2020). Monitoring changes in depression severity using wearable and mobile sensors. Front Psychiatry.

[16] Shah RV, Grennan G, Zafar-Khan M, Alim F, Dey S, Ramanathan D, Mishra J (2021). Personalized machine learning of depressed mood using wearables. Transl Psychiatry.

[17] Wang R, Wang W, daSilva A, Huckins JF, Kelley WM, Heatherton TF, Campbell AT (2018). Tracking depression dynamics in college students using mobile phone and wearable sensing. Proc ACM Interact Mob Wearable Ubiquitous Technol.

[18] Moshe I, Terhorst Y, Opoku Asare K, Sander LB, Ferreira D, Baumeister H, Mohr DC, Pulkki-Råback L (2021). Predicting symptoms of depression and anxiety using smartphone and wearable data. Front Psychiatry.

[19] Rykov Y, Thach TQ, Bojic I, Christopoulos G, Car J (2021). Digital biomarkers for depression screening with wearable devices: cross-sectional study with machine learning modeling. JMIR Mhealth Uhealth.

[20] Sarhaddi F, Azimi I, Niela-Vilen H, Axelin A, Liljeberg P, Rahmani AM (2023). Maternal social loneliness detection using passive sensing through continuous monitoring in everyday settings: longitudinal study. JMIR Form Res.

[21] Faherty LJ, Hantsoo L, Appleby D, Sammel MD, Bennett IM, Wiebe DJ (2017). Movement patterns in women at risk for perinatal depression: use of a mood-monitoring mobile application in pregnancy. J Am Med Inform Assoc.

[22] Shea AK, Kamath MV, Fleming A, Streiner DL, Redmond K, Steiner M (2008). The effect of depression on heart rate variability during pregnancy. A naturalistic study. Clin Auton Res.

[23] Lee K, Kwon H, Lee B, Lee G, Lee JH, Park YR, Shin SY (2018). Effect of self-monitoring on long-term patient engagement with mobile health applications. PLoS One.

[24] Denny JC, Rutter JL, Goldstein DB, Philippakis A, Smoller JW, Jenkins G, Dishman E, All of Us Research Program Investigators (2019). The "All of Us" research program. N Engl J Med.

[25] Bender CG, Hoffstot JC, Combs BT, Hooshangi S, Cappos J (2017). Measuring the fitness of fitness trackers. Proceedings of the 2017 IEEE Sensors Applications Symposium.

[26] Jooma S(E Data and statistics dissemination policy. All of Us Research Program.

[27] Hripcsak G, Duke JD, Shah NH, Reich CG, Huser V, Schuemie MJ, Suchard MA, Park RW, Wong IC, Rijnbeek PR, van der Lei J, Pratt N, Norén GN, Li YC, Stang PE, Madigan D, Ryan PB (2015). Observational health data sciences and informatics (OHDSI): opportunities for observational researchers. Stud Health Technol Inform.

[28] Jones SE, Bradwell KR, Chan LE, McMurry JA, Olson-Chen C, Tarleton J, Wilkins KJ, Ly V, Ljazouli S, Qin Q, Faherty EG, Lau YK, Xie C, Kao YH, Liebman MN, Mariona F, Challa AP, Li L, Ratcliffe SJ, Haendel MA, Patel RC, Hill EL, N3C Consortium (2023). Who is pregnant? Defining real-world data-based pregnancy episodes in the National COVID Cohort Collaborative (N3C). JAMIA Open.

[29] Jukic AM, Baird DD, Weinberg CR, McConnaughey DR, Wilcox AJ (2013). Length of human pregnancy and contributors to its natural variation. Hum Reprod.

[30] Goodman JH (2004). Postpartum depression beyond the early postpartum period. J Obstet Gynecol Neonatal Nurs.

[31] Horowitz JA, Goodman J (2004). A longitudinal study of maternal postpartum depression symptoms. Res Theory Nurs Pract.

[32] Amit G, Girshovitz I, Marcus K, Zhang Y, Pathak J, Bar V, Akiva P (2021). Estimation of postpartum depression risk from electronic health records using machine learning. BMC Pregnancy Childbirth.

[33] ACOG redesigns postpartum care. American College of Obstetricians and Gynecologists.

[34] Liberto TL (2012). Screening for depression and help-seeking in postpartum women during well-baby pediatric visits: an integrated review. J Pediatr Health Care.

[35] Herring SJ, Rich-Edwards JW, Oken E, Rifas-Shiman SL, Kleinman KP, Gillman MW (2008). Association of postpartum depression with weight retention 1 year after childbirth. Obesity (Silver Spring).

[36] Bent B, Goldstein BA, Kibbe WA, Dunn JP (2020). Investigating sources of inaccuracy in wearable optical heart rate sensors. NPJ Digit Med.

[37] Fuller D, Colwell E, Low J, Orychock K, Tobin MA, Simango B, Buote R, Van Heerden D, Luan H, Cullen K, Slade L, Taylor NG (2020). Reliability and validity of commercially available wearable devices for measuring steps, energy expenditure, and heart rate: systematic review. JMIR Mhealth Uhealth.

[38] Master H, Annis J, Huang S, Beckman JA, Ratsimbazafy F, Marginean K, Carroll R, Natarajan K, Harrell FE, Roden DM, Harris P, Brittain EL (2022). Association of step counts over time with the risk of chronic disease in the All of Us research program. Nat Med.

[39] Bates D, Mächler M, Bolker B, Walker S (2015). Fitting linear mixed-effects models using lme4. J Stat Soft.

[40] Kuznetsova A, Brockhoff PB, Christensen RH (2017). Package: tests in linear mixed effects models. J Stat Soft.

[41] Lüdecke D, Ben-Shachar M, Patil I, Waggoner P, Makowski D (2021). performance: an R package for assessment, comparison and testing of statistical models. J Open Source Softw.

[42] English P The its.analysis R package – modelling short time series data. Social Science Research Network.

[43] Abdi H, Williams LJ, Lane DM (2010). Tukey's honestly significant difference (HSD). Encyclopedia of Research Design.

[44] Mughal S, Azhar Y, Siddiqui W Postpartum depression. StatPearls.

[45] Machado-Vieira R, Baumann J, Wheeler-Castillo C, Latov D, Henter ID, Salvadore G, Zarate CA (2010). The timing of antidepressant effects: a comparison of diverse pharmacological and somatic treatments. Pharmaceuticals (Basel).

[46] Abd-Alrazaq A, AlSaad R, Aziz S, Ahmed A, Denecke K, Househ M, Farooq F, Sheikh J (2023). Wearable artificial intelligence for anxiety and depression: scoping review. J Med Internet Res.

[47] Bradley AP (1997). The use of the area under the ROC curve in the evaluation of machine learning algorithms. Pattern Recognit.

[48] Cohen J (2016). A coefficient of agreement for nominal scales. Educ Psychol Meas.

[49] Hand DJ, Till RJ (2001). A simple generalisation of the area under the ROC curve for multiple class classification problems. Mach Learn.

[50] Kuhn M (2008). Building predictive models in R using the caret package. J Stat Soft.

[51] Molnar C, Casalicchio G, Bischl B (2018). iml: an R package for interpretable machine learning. J Open Source Softw.

[52] Lundberg SM, Erion GG, Lee SI Consistent individualized feature attribution for tree ensembles. https://arxiv.org/abs/1802.03888.

[53] Chaibub Neto E, Pratap A, Perumal TM, Tummalacherla M, Snyder P, Bot BM, Trister AD, Friend SH, Mangravite L, Omberg L (2019). Detecting the impact of subject characteristics on machine learning-based diagnostic applications. NPJ Digit Med.

[54] Belmaker RH, Agam G (2008). Major depressive disorder. N Engl J Med.

[55] Doryab A, Min JK, Wiese J, Zimmerman J, Hong JI (2014). Detection of behavior change in people with depression. Proceedings of the 28th Conference on Artificial Intelligence.

[56] Heidemann BH, McClure JH (2003). Changes in maternal physiology during pregnancy. BJA CEPD Rev.

[57] Soma-Pillay P, Nelson-Piercy C, Tolppanen H, Mebazaa A (2016). Physiological changes in pregnancy. Cardiovasc J Afr.

[58] Ling CX, Huang J, Zhang H (2003). AUC: a better measure than accuracy in comparing learning algorithms. Proceedings of the 16th Conference of the Canadian Society for Computational Studies of Intelligence.

[59] Lundberg SM, Lee SI A unified approach to interpreting model predictions.

[60] Fisher A, Rudin C, Dominici F All models are wrong, but many are useful: learning a variable's importance by studying an entire class of prediction models simultaneously. https://arxiv.org/abs/1801.01489.

[61] Attanasio LB, Ranchoff BL, Cooper MI, Geissler KH (2022). Postpartum visit attendance in the United States: a systematic review. Womens Health Issues.

[62] Guedeney N, Fermanian J, Guelfi JD, Kumar RC (2000). The Edinburgh Postnatal Depression Scale (EPDS) and the detection of major depressive disorders in early postpartum: some concerns about false negatives. J Affect Disord.

[63] Levis B, Negeri Z, Sun Y, Benedetti A, Thombs BD, DEPRESsion Screening Data (DEPRESSD) EPDS Group (2020). Accuracy of the Edinburgh Postnatal Depression Scale (EPDS) for screening to detect major depression among pregnant and postpartum women: systematic review and meta-analysis of individual participant data. BMJ.

[64] Drysdale AT, Grosenick L, Downar J, Dunlop K, Mansouri F, Meng Y, Fetcho RN, Zebley B, Oathes DJ, Etkin A, Schatzberg AF, Sudheimer K, Keller J, Mayberg HS, Gunning FM, Alexopoulos GS, Fox MD, Pascual-Leone A, Voss HU, Casey B, Dubin MJ, Liston C (2017). Resting-state connectivity biomarkers define neurophysiological subtypes of depression. Nat Med.

[65] Halaris A (2011). A primary care focus on the diagnosis and treatment of major depressive disorder in adults. J Psychiatr Pract.

[66] Institute of Medicine (US) Forum on Drug Discovery, Development, and Translation (2010). Transforming Clinical Research in the United States: Challenges and Opportunities: Workshop Summary.

[67] Dennis CL, Ross LE (2006). Depressive symptomatology in the immediate postnatal period: identifying maternal characteristics related to true- and false-positive screening scores. Can J Psychiatry.

[68] Li S, Yang Z, Yao M, Shen Y, Zhu H, Jiang Y, Ji Y, Yin J (2022). Exploration for biomarkers of postpartum depression based on metabolomics: a systematic review. J Affect Disord.

[69] Poppitt SD, Prentice AM, Goldberg GR, Whitehead RG (1994). Energy-sparing strategies to protect human fetal growth. Am J Obstet Gynecol.

[70] Tinius RA, Yoho K, Blankenship MM, Maples JM (2021). Postpartum metabolism: how does it change from pregnancy and what are the potential implications?. Int J Womens Health.

[71] Crozier SR, Robinson SM, Borland SE, Godfrey KM, Cooper C, Inskip HM, Study Group S (2009). Do women change their health behaviours in pregnancy? Findings from the Southampton women's survey. Paediatr Perinat Epidemiol.

[72] Lindqvist M, Lindkvist M, Eurenius E, Persson M, Mogren I (2017). Change of lifestyle habits - motivation and ability reported by pregnant women in northern Sweden. Sex Reprod Healthc.

[73] Zinsser LA, Stoll K, Wieber F, Pehlke-Milde J, Gross MM (2020). Changing behaviour in pregnant women: a scoping review. Midwifery.

[74] How does my Fitbit device calculate my daily activity?. Fitbit Help Center.

[75] Sharma AJ, Bulkley JE, Stoneburner AB, Dandamudi P, Leo M, Callaghan WM, Vesco KK (2021). Bias in self-reported prepregnancy weight across maternal and clinical characteristics. Matern Child Health J.

[76] Fadzil F, Shamsuddin K, Wan Puteh SE, Mohd Tamil A, Ahmad S, Abdul Hayi NS, Abdul Samad A, Ismail R, Ahmad Shauki NI (2018). Predictors of postpartum weight retention among urban Malaysian mothers: a prospective cohort study. Obes Res Clin Pract.

[77] Pettersson CB, Zandian M, Clinton D (2016). Eating disorder symptoms pre- and postpartum. Arch Womens Ment Health.

[78] Fowles ER, Stang J, Bryant M, Kim S (2012). Stress, depression, social support, and eating habits reduce diet quality in the first trimester in low-income women: a pilot study. J Acad Nutr Diet.

[79] Easterling TR, Benedetti TJ, Schmucker BC, Millard SP (1990). Maternal hemodynamics in normal and preeclamptic pregnancies: a longitudinal study. Obstet Gynecol.

[80] Odendaal H, Groenewald C, Myers MM, Fifer WP (2018). Maternal heart rate patterns under resting conditions in late pregnancy. Trends Res.

[81] Thornburg KL, Jacobson SL, Giraud GD, Morton MJ (2000). Hemodynamic changes in pregnancy. Semin Perinatol.

[82] Hu S, Tucker L, Wu C, Yang L (2020). Beneficial effects of exercise on depression and anxiety during the COVID-19 pandemic: a narrative review. Front Psychiatry.

[83] Zhou ZH (2021). Machine Learning.

[84] Garcia-Ceja E, Riegler M, Jakobsen P, Torresen J, Nordgreen T, Oedegaard KJ, Fasmer OB (2018). Motor activity based classification of depression in unipolar and bipolar patients. Proceedings of the IEEE 31st International Symposium on Computer-Based Medical Systems.

[85] Jacobson NC, Weingarden H, Wilhelm S (2019). Digital biomarkers of mood disorders and symptom change. NPJ Digit Med.

[86] Opoku Asare K, Moshe I, Terhorst Y, Vega J, Hosio S, Baumeister H, Pulkki-Råback L, Ferreira D (2022). Mood ratings and digital biomarkers from smartphone and wearable data differentiates and predicts depression status: a longitudinal data analysis. Pervasive and Mobile Computing.

[87] Apostolopoulos M, Hnatiuk JA, Maple JL, Olander EK, Brennan L, van der Pligt P, Teychenne M (2021). Influences on physical activity and screen time amongst postpartum women with heightened depressive symptoms: a qualitative study. BMC Pregnancy Childbirth.

[88] Glinoer D (1999). What happens to the normal thyroid during pregnancy?. Thyroid.

[89] Jardí C, Aparicio E, Bedmar C, Aranda N, Abajo S, March G, Basora J, Arija V, The Eclipses Study Group (2019). Food consumption during pregnancy and post-partum. ECLIPSES study. Nutrients.

[90] La Marca-Ghaemmaghami P, Ehlert U (2015). Stress During Pregnancy. Eur Psychol.

[91] Lain KY, Catalano PM (2007). Metabolic changes in pregnancy. Clin Obstet Gynecol.

[92] Hawkins DM (2004). The problem of overfitting. J Chem Inf Comput Sci.

[93] Spoont MR, Depue RA, Krauss SS (1991). Dimensional measurement of seasonal variation in mood and behavior. Psychiatry Res.

[94] Chen T, Guestrin C (2016). XGBoost: a scalable tree boosting system. Proceedings of the 22nd ACM SIGKDD International Conference on Knowledge Discovery and Data Mining.

[95] Home page. OpenAI.

