# Supplementary material for: Harnessing Consumer Wearable Digital Biomarkers for Individualized Recognition of Postpartum Depression Using the All of Us Research Program Data Set: Cross-Sectional Study
Source: JMIR Mhealth Uhealth. 2024 May 2;12:e54622. doi: 10.2196/54622 (PMC11099816; doi:10.2196/54622)
Supplement: Multimedia Appendix 2 [file mhealth_v12i1e54622_app2.docx]

**Multimedia Appendix 2. Supplementary results, tables, and figures.**

**Table S1: Digital biomarkers measured by the Fitbit available in the *All of Us* Research Program (AoURP) dataset v6.**

| **Name** | **Category** | **Description** |
| --- | --- | --- |
| Average HR | Heart rate | Mean heart rate per date |
| HR SD | Heart rate | Heart rate’s standard deviation per date |
| Minimum HR | Heart rate | Minimum heart rate per date |
| Q1 HR | Heart rate | Heart rate’s first quartile per date |
| Median HR | Heart rate | Median heart rate per date |
| Q3 HR | Heart rate | Heart rate’s third quartile per date |
| Maximum HR | Heart rate | Maximum heart rate per date |
| Sum steps | Activity | Sum of steps per date |
| Activity calories | Activity | Sum of calories burned when not at rest per date |
| Calories BMR | Activity | Sum of calories burned at rest per date to maintain vital body functions, such as breathing, blood circulation, and heartbeat |
| Calories out | Activity | Sum of total calories burned per date |
| Fairly active minutes | Activity | Sum of fairly active minutes per date |
| Lightly active minutes | Activity | Sum of lightly active minutes per date |
| Marginal calories | Activity | Difference in calories burned at rest and during activity per date |
| Sedentary minutes | Activity | Sum of sedentary minutes per date |
| Very active minutes | Activity | Sum of very active minutes per date |

**Table S2: Random effect *P* value from linear mixed effects model with person ID as the random effect.**

| **Digital biomarker** | **Degrees of freedom** | ***P*** |
| --- | --- | --- |
| Average HR | 1 | <.001 |
| HR SD | 1 | <.001 |
| Minimum HR | 1 | <.001 |
| Q1 HR | 1 | <.001 |
| Median HR | 1 | <.001 |
| Q3 HR | 1 | <.001 |
| Maximum HR | 1 | <.001 |
| Sum steps | 1 | <.001 |
| Activity calories | 1 | <.001 |
| Calories BMR | 1 | <.001 |
| Calories out | 1 | <.001 |
| Fairly active minutes | 1 | <.001 |
| Lightly active minutes | 1 | <.001 |
| Marginal calories | 1 | <.001 |
| Sedentary minutes | 1 | <.001 |
| Very active minutes | 1 | <.001 |

*Statistical tests were run at a significance level of .05.

**Table S3: Percentage of women with a significant difference between time periods from the interrupted time series analysis (ITSA).**

| **Digital biomarker** | **Percentage significant** |
| --- | --- |
| Average HR | 65-70 |
| HR SD | 75-80 |
| Minimum HR | 65-70 |
| Q1 HR | 70-75 |
| Median HR | 65-70 |
| Q3 HR | 70-75 |
| Maximum HR | 65-70 |
| Sum steps | 65-70 |
| Activity calories | 75-80 |
| Calories BMR | 50-55 |
| Calories out | 65-70 |
| Fairly active minutes | 75-80 |
| Lightly active minutes | 75-80 |
| Marginal calories | 75-80 |
| Sedentary minutes | 60-65 |
| Very active minutes | 60-65 |

*Statistical tests were run at a significance level of .05.

**Table S4: Net direction of digital biomarkers between pairs of time periods.**

| **Group** | **Direction** | **Digital biomarker** |
| --- | --- | --- |
| PPD-prepregnancy | Positive | Sedentary minutes |
| PPD-prepregnancy | Negative | HR SD, Sum steps, Activity calories, Calories out, Fairly active minutes, Marginal calories, Very active minutes |
| PPD-prepregnancy | Not significant | Average HR, Minimum HR, Q1 HR, Median HR, Q3 HR, Maximum HR, Calories BMR, Lightly active minutes |
| PPD-pregnancy | Negative | Average HR, Minimum HR, Q1 HR, Median HR, Q3 HR, Maximum HR, Sum steps, Fairly active minutes, Marginal calories, Very active minutes |
| PPD-pregnancy | Not significant | HR SD, Activity calories, Calories BMR, Calories out, Lightly active minutes, Sedentary minutes |
| PPD-postpartum | Positive | None |
| PPD-postpartum | Negative | None |
| PPD-postpartum | Not significant | Average HR, HR SD, Minimum HR, Q1 HR, Median HR, Q3 HR, Maximum HR, Sum steps, Activity calories, Calories BMR, Calories out, Fairly active minutes, Lightly active minutes, Marginal calories, Sedentary minutes, Very active minutes |
| Postpartum-prepregnancy | Positive | Sedentary minutes |
| Postpartum-prepregnancy | Negative | HR SD, Maximum HR, Sum steps, Activity calories, Fairly active minutes, Marginal calories, Very active minutes |
| Postpartum-prepregnancy | Not significant | Average HR, Minimum HR, Q1 HR, Median HR, Q3 HR, Calories BMR, Calories out, Lightly active minutes |
| Postpartum-pregnancy | Positive | Sedentary minutes |
| Postpartum-pregnancy | Negative | Maximum HR, Sum steps, Fairly active minutes, Very active minutes |
| Postpartum-pregnancy | Not significant | Average HR, HR SD, Minimum HR, Q1 HR, Median HR, Q3 HR, Activity calories, Calories BMR, Calories out, Lightly active minutes, Marginal calories |
| Pregnancy-prepregnancy | Positive | Average HR, Minimum HR, Q1 HR, Median HR, Q3 HR |
| Pregnancy-prepregnancy | Negative | HR SD, Sum steps, Activity calories, Fairly active minutes, Lightly active minutes, Marginal calories, Very active minutes |
| Pregnancy-prepregnancy | Not significant | Maximum HR, Calories BMR, Calories out, Sedentary minutes |

*Statistical tests were run at a significance level of .05.

**Figure S1: Individualized random forest (RF) models exhibited a strong performance for identifying all four time periods.**

**
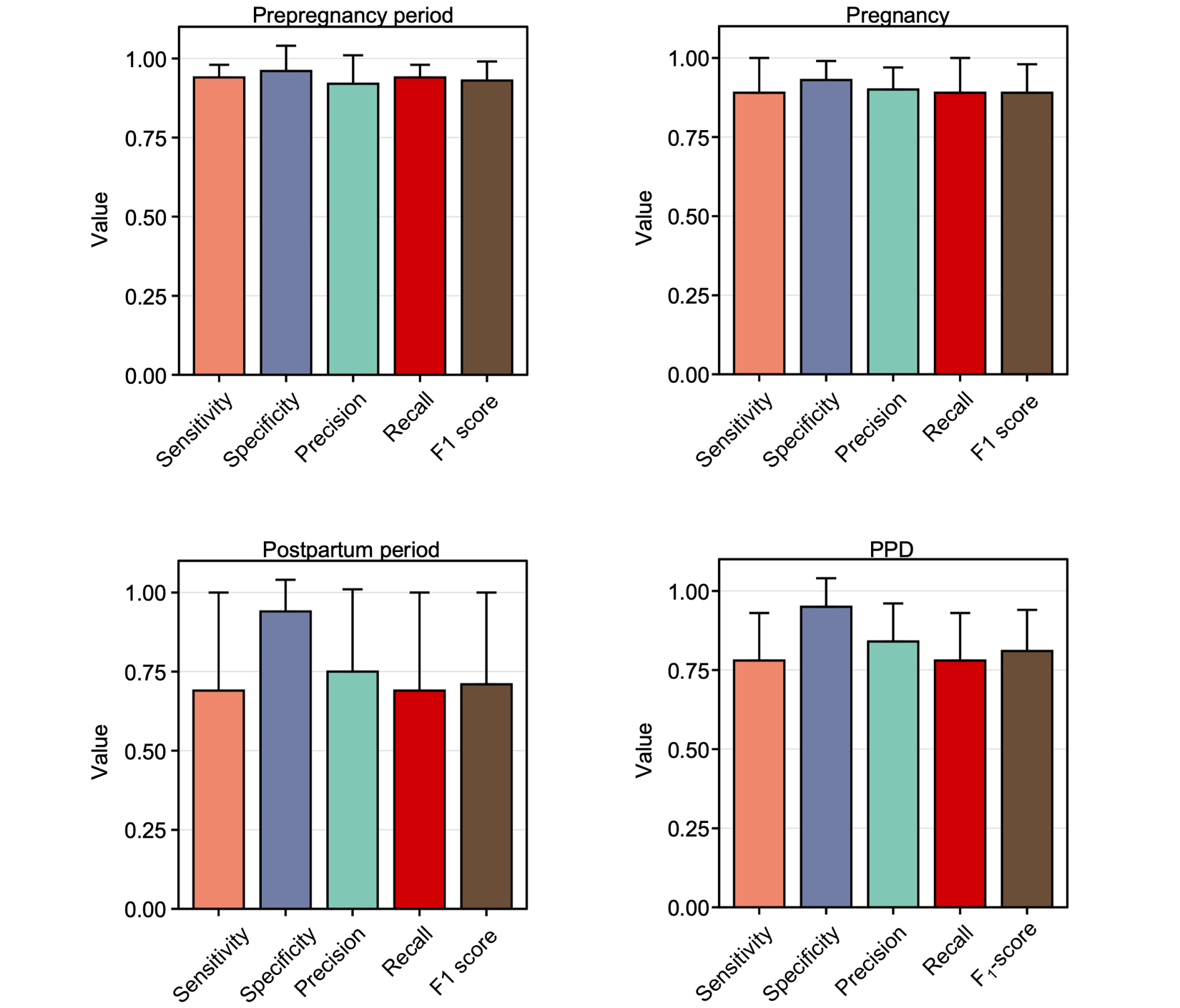
**

*The sensitivity, specificity, precision, recall, and F_1_-score across individual RF models for the prepregnancy (top left), pregnancy (top right), postpartum (bottom left), and PPD (bottom right) periods.*

***Figure S1 legend:*** *Individualized RF models exhibited robust performance in terms of sensitivity, specificity, precision, recall, and F_1_-score for recognizing the prepregnancy, pregnancy, postpartum, and PPD time periods.*

*Data are expressed as mean±SD.*

**Figure S2: Prior history of depression before or during pregnancy did not impact model performance for recognizing the PPD time period.**

**
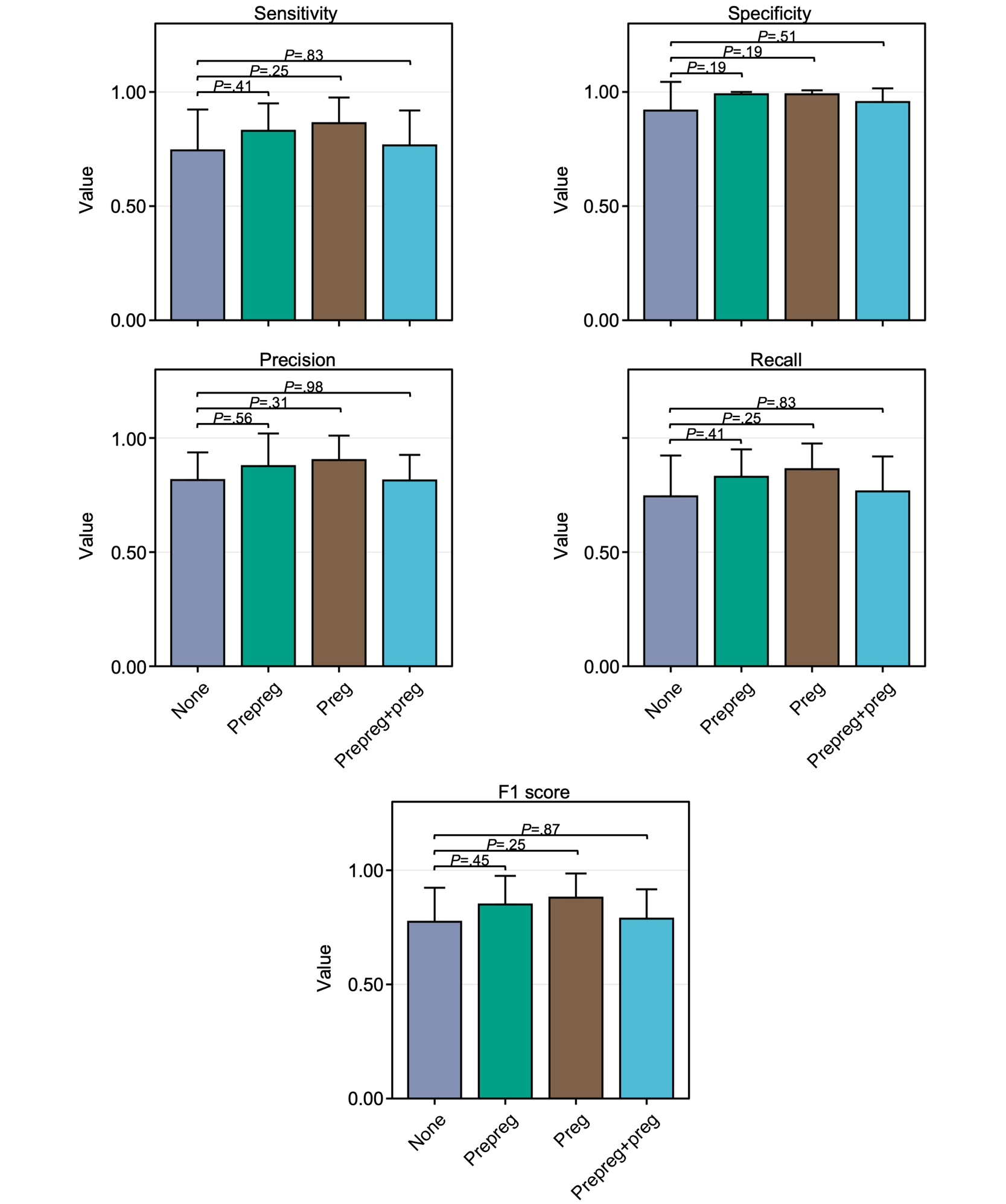
**

***Figure S2 legend:*** *The sensitivity (top left), specificity (top right), precision (middle left), recall (middle right), and F_1_-score(bottom middle) of individualized machine learning (ML) models in women from the PPD cohort with 1) no prior history of depression (None), 2) history prepregnancy (Prepreg), 3) history during pregnancy (Preg), or 4) history of both prepregnancy and during pregnancy (Prepreg+preg). Among women in the PPD cohort, the average sensitivity, specificity, precision, recall, and F_1_-score displayed no variation across those with no history of depression, history of depression prior to pregnancy, history of depression during pregnancy, or history of depression both prior to and during pregnancy. Data are expressed as mean±SD.*

**Figure S3: SHapley Additive exPlanations (SHAP) beeswarm plots were used for variable importance in individualized ML models.**

**
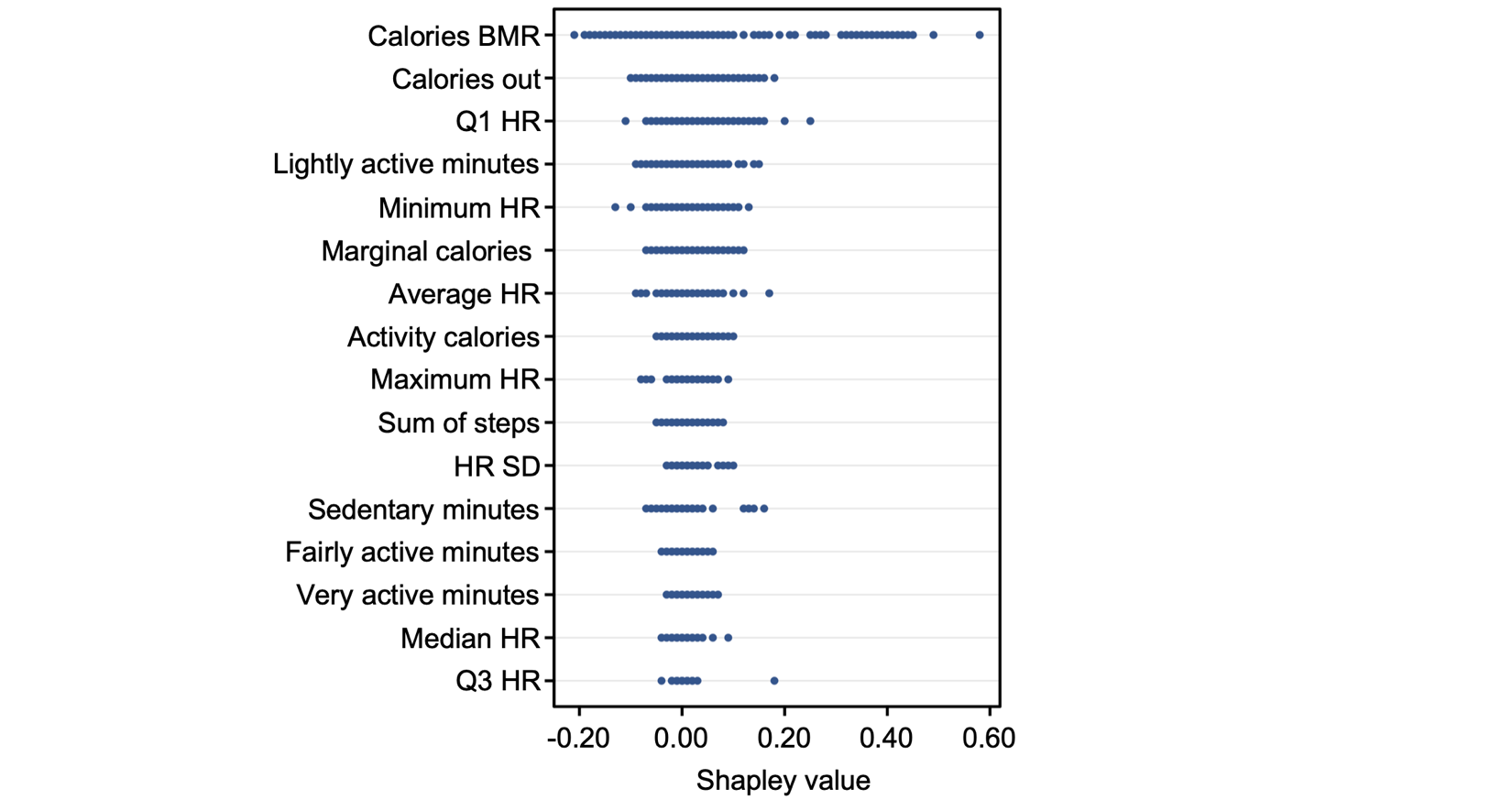
**

***Figure S3 legend:*** *An example of a beeswarm SHapley Additive exPlanations (SHAP) plot for assessing variable importance in individualized ML models.*

*An example SHAP beeswarm plot showcasing the ranking of digital biomarkers according to their average Absolute Shapley values within an individualized model for predicting the PPD time period. In this example, the five most predictive features for the PPD time period were calories BMR, calories out, Q1 HR, lightly active minutes, and minimum HR.*

**Figure S4: An example of SHAP dependence plots assessing the relationship between digital biomarkers and PPD.**

**
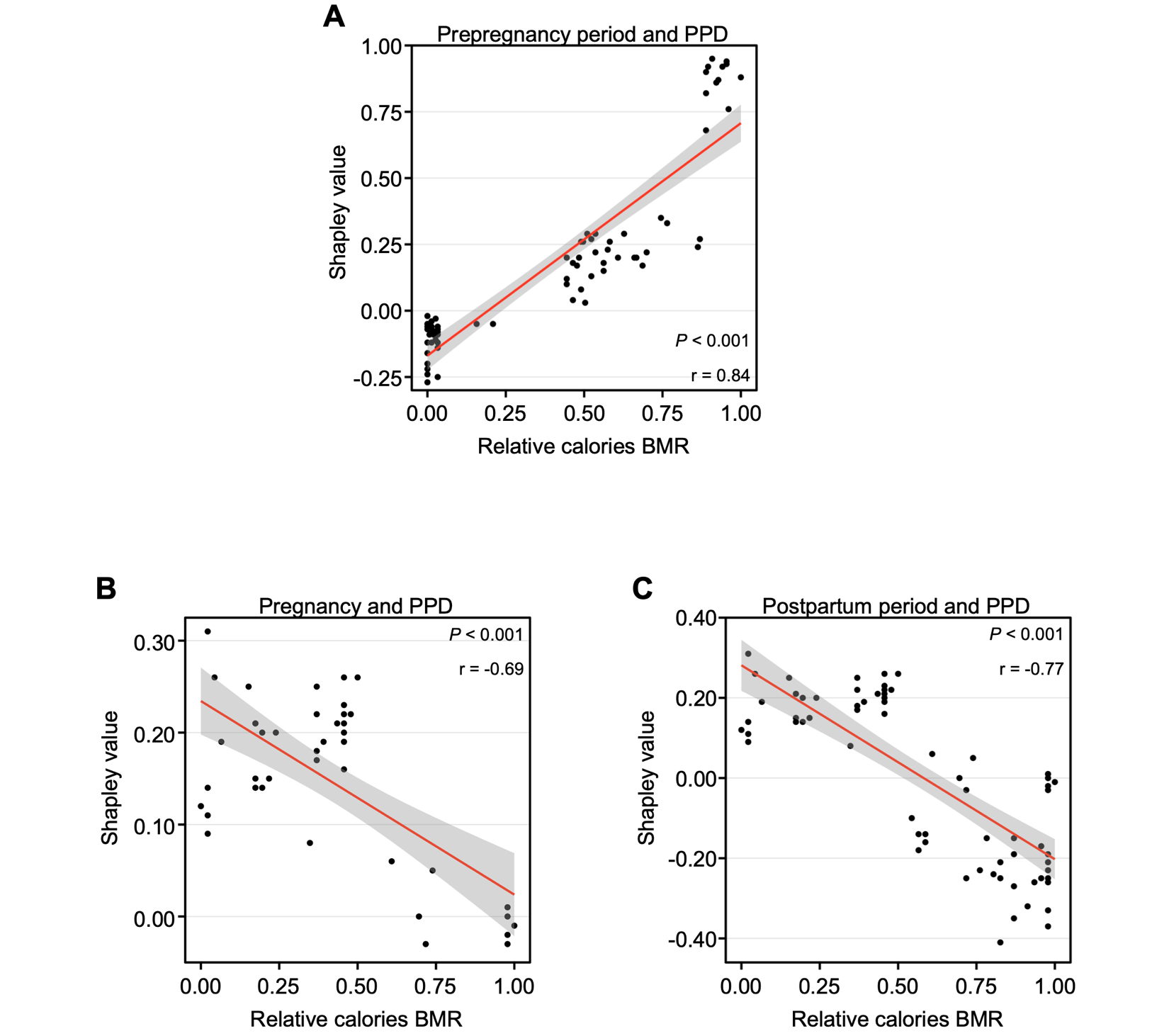
**

***A:*** *An example of a SHAP dependence plot during prepregnancy/PPD time periods.*

***B:*** *An example of a SHAP dependence plot during pregnancy/PPD time periods.*

***C:*** *An example of a SHAP dependence plot during postpartum/PPD time periods.*

***Figure S4 legend:*** *Example SHAP dependence plots from individualized models demonstrated a correlation between Shapley values and actual values of calories BMR between the prepregnancy/PPD time periods in addition to a negative correlation during the pregnancy/PPD and postpartum/PPD time periods. This process was repeated for each individual to calculate the percentage of women with a significant correlation. Of those with a significant correlation, the percentage of women with a positive or negative correlation between Shapley values and actual values of the digital biomarker of interest was calculated.*

**Figure S5: The relationship between digital biomarkers provided insights into PPD classification in individualized N-of-1 ML models.**

**
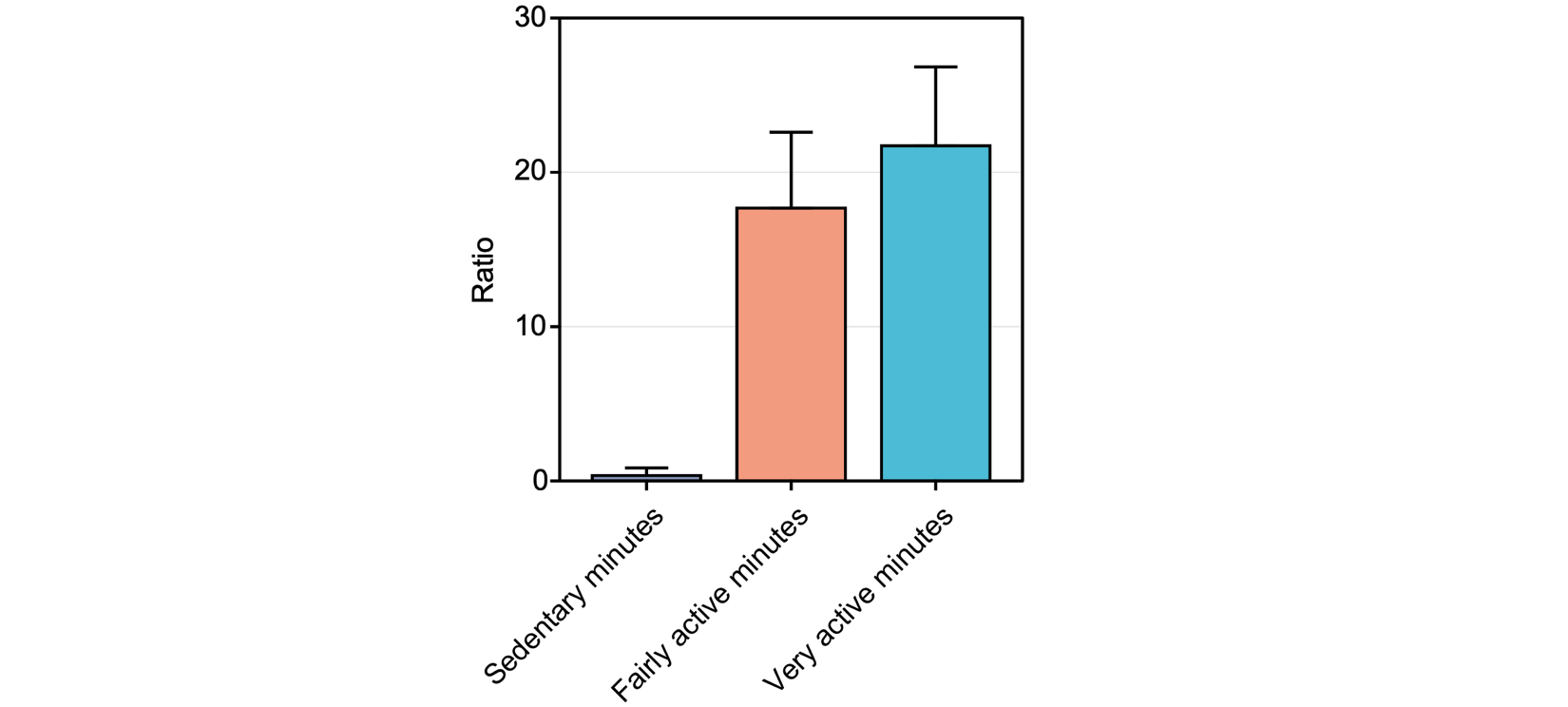
**

***Figure S5 legend:*** *The ratio of lightly active minutes to sedentary minutes, fairly active minutes, and very active minutes across all individuals during the pregnancy and PPD time periods. The ratio of lightly active minutes to very active minutes surpassed that of lightly active minutes to fairly active minutes, which in turn is greater than the ratio of lightly active minutes to sedentary minutes. Data are expressed as mean±SD.*

**Supplementary Results - Healthcare utilization:** ACOG recommends that women in the postpartum period attend at least one visit within the first six weeks following delivery; however, women diagnosed with PPD experience variations in healthcare-seeking behavior, leading to delayed identification of the condition [33,34]. To investigate this impact within our cohort of women with PPD, we evaluated the frequency of postpartum visits among women in both the PPD and non-PPD cohorts, where our findings demonstrated no statistically significant difference in the median (and IQR) number of visits (*P*=.52) between women in the PPD cohort (median=4.5 visits, IQR=9.75) and those in the non-PPD cohort (median=4 visits, IQR=4). We additionally examined the proportion of women in each cohort who attended at least one visit within the first six weeks of the postpartum period, following the recommended guidelines by ACOG [33]. Our findings indicated approximately 80% and 86% of women in the PPD and non-PPD cohorts adhered to this guideline, which was not significantly different (*P*=.73) (Table S5).

**Table S5: Postpartum visits among women in the PPD and non-PPD cohorts.**

|  | **PPD cohort** | | **Non-PPD cohort** | |  |
| --- | --- | --- | --- | --- | --- |
|  | **Median** | **IQR** | **Median** | **IQR** | ***P*** |
| Number of visits | 4.5 | 9.75 | 4 | 4 | .52 |
|  | **Percent** | | **Percent** | |  |
| Women with a postpartum visit within six weeks | 80 | | 86 | | .73 |

IQR = interquartile range

**Supplementary Results - Accuracy of self-reported weight:** Having seen that calories BMR was the most predictive feature of PPD, we aimed to compare the accuracy of self-reported weight in our PPD and non-PPD cohorts. This was accomplished by determining the percent difference between the two closest self-reported and the gold-standard weight measurements. The percent difference between self-reported weight and gold-standard weight was minimal, with the PPD cohort showing a median of 1.87% (IQR=0.67) and the non-PPD cohort displaying a median of 1.02% (IQR=1.85) (Table S6). Moreover, the percent differences between the PPD and non-PPD cohorts were not significantly different (*P*=.60) (Table S6).

**Table S6: Comparing self-reported and gold-standard weight measurements among women in the PPD and non-PPD cohorts.**

|  | **Self-reported** | | **Gold-standard** | |  |
| --- | --- | --- | --- | --- | --- |
| **Cohort** | **Median** | **IQR** | **Median** | **IQR** | ***P*** |
| PPD | 78.35 | 14.70 | 78.22 | 15.25 | .83 |
| Non-PPD | 71.20 | 17.20 | 72.34 | 17.61 | .77 |

**Supplementary Results - Weight difference across time periods in PPD and non-PPD cohorts:** After observing the predictive value of calories BMR in predicting PPD in individualized ML models and observing the relationship from SHAP dependence plots, we aimed to explore the association between weight and PPD. This exploration stemmed from calories BMR being calculated based on age, sex, height, and weight. We first confirmed the positive relationship between calories BMR and weight (*r*=0.88, *P*<.001). Next, we constructed linear-mixed effects models to compare the difference in weight across time periods for women in our PPD and non-PPD cohorts. Relative to the PPD time period (for women in the PPD cohort), the results revealed the estimates for prepregnancy=-1.75 kg (standard error [SE]=1.22, *P*=.15), pregnancy=3.21 kg (SE=0.98, *P*=.001), and postpartum=6.66 kg (SE=1.73, *P*<.001) (Table S7). These data suggest no significant change in weight during prepregnancy, an increase of 3.2 kg during pregnancy, and an increase of 6.66 kg during the postpartum period when compared to the PPD time period. In contrast, in the non-PPD cohort, the estimates relative to the PPD-equivalent time period were prepregnancy=-6.28 kg (SE=0.94, *P*<.001), pregnancy=2.19 kg (SE=0.81, *P*=.01), and postpartum=0.16 kg (SE=1.50, *P*=.91) (Table S7). These results suggest a 6.3kg decrease during prepregnancy, a 2.19 kg increase, and no significant difference during the postpartum period compared to the PPD-equivalent time period (Tables S7 and S8).

**Table S7: The estimated difference in weight among women in the PPD cohort across time periods.**

|  | **PPD cohort** | | | **Non-PPD cohort** | | |
| --- | --- | --- | --- | --- | --- | --- |
| **Time period** | Estimate | SE | *P* | Estimate | SE | *P* |
| Intercept* | 82.53 | 4.91 | <.001 | 74.42 | 2.18 | <.001 |
| Prepregnancy | -1.75 | 1.22 | .15 | -6.28 | 0.94 | <.001 |
| Pregnancy | 3.21 | 0.98 | <.001 | 2.19 | 0.81 | .007 |
| Postpartum | 6.66 | 1.73 | <.001 | 0.16 | 1.50 | .91 |

* Intercept in the PPD cohort = PPD time period, while intercept for women in the non-PPD cohort = PPD-equivalent time period; SE = standard error

**Table S8: The estimated mean weight among women in the PPD cohort across time periods.**

|  | **PPD cohort** | | | | **Non-PPD cohort** | | | |
| --- | --- | --- | --- | --- | --- | --- | --- | --- |
| **Time period** | emmean | SE | Lower CL | Upper CL | emmean | SE | Lower CL | Upper CL |
| Prepregnancy | 80.78 | 4.13 | 71.79 | 89.77 | 68.14 | 2.19 | 63.71 | 72.57 |
| Pregnancy | 85.73 | 4.08 | 76.80 | 94.67 | 76.62 | 2.14 | 72.28 | 80.97 |
| Postpartum | 89.18 | 4.32 | 79.95 | 98.42 | 74.59 | 2.47 | 69.65 | 76.53 |
| PPD (or PPD-eq) | 82.53 | 4.09 | 73.58 | 91.47 | 74.42 | 2.18 | 70.01 | 78.83 |

*PPD-eq = PPD-equivalent time period which represents the fourth corresponding time period in women without PPD; SE = standard error; CL = confidence level

**Supplementary Results - The relationship between weight, calories BMR, and PPD:** We further sought to evaluate whether the relationships identified in the SHAP dependence plots involving calories BMR and PPD (in comparison to other time periods) would indicate a correlation with weight. First, we hypothesized that the 60-65% of women displaying a positive relationship in SHAP dependence plots, signifying an increased calories BMR during the PPD period compared to prepregnancy, suggested weight gain during PPD relative to prepregnancy. To probe this hypothesis, we filtered data on the 60-65% of women in the PPD cohort exhibiting this positive relationship and constructed a linear mixed effects model with person ID as the random effect. The results demonstrated an increase in body weight during the PPD time period relative to prepregnancy (estimate=-6.63 kg, SE=1.51, *P*<.001 [note PPD is the reference time period]) (Table S9). This result supports the hypothesis, indicating an estimated increase of 6.63 kg during PPD compared to the prepregnancy period. The same method was applied to examine the association between body weight among the 85-90% of women displaying a negative correlation between calories BMR during the postpartum and PPD time periods, suggesting a decline in calories BMR linked to PPD relative to the postpartum period. Similarly, the data showed that women in the PPD time period exhibit a weight loss of 7.82 kg relative to the postpartum period (SE=1.83, *P*<.001), suggesting that changes in calories BMR are indicative of weight changes (Table S10).

**Table S9: The estimated difference in weight among women in the PPD cohort who displayed a positive relationship between calories BMR and PPD relative to prepregnancy.**

| **Time period** | **Estimate** | **SE** | ***P*** |
| --- | --- | --- | --- |
| Intercept* | 78.48 | 4.25 | < .001 |
| Prepregnancy | -6.63 | 1.51 | < .001 |

* Intercept = PPD time period; SE = standard error

**Table S10: The estimated difference in weight among women in the PPD cohort who displayed a negative relationship between calories BMR and PPD relative to the postpartum period.**

| **Time period** | **Estimate** | **SE** | ***P*** |
| --- | --- | --- | --- |
| Intercept* | 76.42 | 3.62 | < .001 |
| Postpartum | 7.82 | 1.83 | < .001 |

* Intercept = PPD time period; SE = standard error

**Supplementary Results - Weight retention:** Prior studies have suggested that women with PPD experience more weight gain/retention compared to those without PPD [35]. Therefore, we sought to answer this question in our PPD and non-PPD cohorts. Surprisingly, the results demonstrated a non-significant difference (*P*=.23) in the number of days it took women in the PPD cohort (median=402.5, IQR=223.5) to return to their prepregnancy weight compared to those in the non-PPD cohort (median=336.5, IQR=57.5) (Table S11). To confirm this observation, we leveraged the availability of calories BMR given its relationship with weight. The outcome was consistent and displayed a similar number of days returning to prepregnancy calories BMR in the PPD (median=300, IQR=43.5) and non-PPD cohorts (median=321.5, IQR=80.25, *P*=0.47) (Table S11).

**Table S11: The number of days for women in the PPD and non-PPD cohorts to return to prepregnancy weight.**

|  | **PPD cohort** | | **Non-PPD cohort** | |  |
| --- | --- | --- | --- | --- | --- |
|  | **Median** | **IQR** | **Median** | **IQR** | ***P*** |
| Number of days (weight measurements) | 402.5 | 223.5 | 336.5 | 57.5 | .23 |
| Number of days (calories BMR) | 300 | 43.5 | 321.5 | 80.25 | .47 |
